# Intracellular calcium stores mediate metaplasticity at hippocampal dendritic spines

**DOI:** 10.1113/JP277726

**Published:** 2019-06-02

**Authors:** Gaurang Mahajan, Suhita Nadkarni

**Affiliations:** ^1^ Indian Institute of Science Education and Research Pune 411 008 India

**Keywords:** Synaptic plasticity, Intracellular calcium stores, Calcium signaling, Metaplasticity, Biophysical modeling

## Abstract

**Key points:**

- Calcium (Ca^2+^) entry mediated by NMDA receptors is considered central to the induction of activity‐dependent synaptic plasticity in hippocampal area CA1; this description does not, however, take into account the potential contribution of endoplasmic reticulum (ER) Ca^2+^ stores.
- The ER has a heterogeneous distribution in CA1 dendritic spines, and may introduce localized functional differences in Ca^2+^ signalling between synapses, as suggested by experiments on metabotropic receptor‐dependent long‐term depression.
- A physiologically detailed computational model of Ca^2+^ dynamics at a CA3–CA1 excitatory synapse characterizes the contribution of spine ER via metabotropic signalling during plasticity induction protocols.
- ER Ca^2+^ release via IP_3_ receptors modulates NMDA receptor‐dependent plasticity in a graded manner, to selectively promote synaptic depression with relatively diminished effect on LTP induction; this may temper further strengthening at the stronger synapses which are preferentially associated with ER‐containing spines.
- Acquisition of spine ER may thus represent a local, biophysically plausible ‘metaplastic switch’ at potentiated CA1 synapses, contributing to the plasticity–stability balance in neural circuits.

**Abstract:**

Long‐term plasticity mediated by NMDA receptors supports input‐specific, Hebbian forms of learning at excitatory CA3–CA1 connections in the hippocampus. There exists an additional layer of stabilizing mechanisms that act globally as well as locally over multiple time scales to ensure that plasticity occurs in a constrained manner. Here, we investigated the role of calcium (Ca^2+^) stores associated with the endoplasmic reticulum (ER) in the local regulation of plasticity at individual CA1 synapses. Our study was spurred by (1) the curious observation that ER is sparsely distributed in dendritic spines, but over‐represented in larger spines that are likely to have undergone activity‐dependent strengthening, and (2) evidence suggesting that ER motility at synapses can be rapid, and accompany activity‐regulated spine remodelling. We constructed a physiologically realistic computational model of an ER‐bearing CA1 spine, and examined how IP_3_‐sensitive Ca^2+^ stores affect spine Ca^2+^ dynamics during activity patterns mimicking the induction of long‐term potentiation and long‐term depression (LTD). Our results suggest that the presence of ER modulates NMDA receptor‐dependent plasticity in a graded manner that selectively enhances LTD induction. We propose that ER may locally tune Ca^2+^‐based plasticity, providing a braking mechanism to mitigate runaway strengthening at potentiated synapses. Our study provides a biophysically accurate description of postsynaptic Ca^2+^ regulation, and suggests that ER in the spine may promote the re‐use of hippocampal synapses with saturated strengths.

## Key points

- Calcium (Ca^2+^) entry mediated by NMDA receptors is considered central to the induction of activity‐dependent synaptic plasticity in hippocampal area CA1; this description does not, however, take into account the potential contribution of endoplasmic reticulum (ER) Ca^2+^ stores.
- The ER has a heterogeneous distribution in CA1 dendritic spines, and may introduce localized functional differences in Ca^2+^ signalling between synapses, as suggested by experiments on metabotropic receptor‐dependent long‐term depression.
- A physiologically detailed computational model of Ca^2+^ dynamics at a CA3–CA1 excitatory synapse characterizes the contribution of spine ER via metabotropic signalling during plasticity induction protocols.
- ER Ca^2+^ release via IP_3_ receptors modulates NMDA receptor‐dependent plasticity in a graded manner, to selectively promote synaptic depression with relatively diminished effect on LTP induction; this may temper further strengthening at the stronger synapses which are preferentially associated with ER‐containing spines.
- Acquisition of spine ER may thus represent a local, biophysically plausible ‘metaplastic switch’ at potentiated CA1 synapses, contributing to the plasticity–stability balance in neural circuits.

## Introduction

Hebbian synaptic plasticity involves activity‐driven changes in synaptic strengths (Hebb, 1949). This form of plasticity is inherently unstable, as a small change in synaptic strength can promote further change in the same direction, and this positive reinforcement can drive synaptic efficacies to either saturate or reduce to a minimum (Abbott & Nelson, 2000; Honnuraiah & Narayanan, 2013; Hobbiss *et al*. 2018). It has long been recognized that Hebbian rules need to be supplemented with additional stabilizing mechanisms to curb runaway plasticity and support stable yet flexible neural circuits (Abbott & Nelson, 2000; Zenke & Gerstner, 2017; Keck *et al*. 2017). The issue of stability is usually addressed within the theoretical Bienenstock–Cooper–Munro (BCM) framework (Bienenstock *et al*. 1982), which posits an adaptive threshold for long‐term potentiation induction that varies as a function of the history of prior activity of the postsynaptic neuron, concurrently affecting all its afferent synapses. There is, however, limited understanding of biophysical mechanisms implementing such a rule (Gold & Bear, 1994; Bear, 1995; Abraham *et al*. 2001; Philpot *et al*. 2003; Xu *et al*. 2009; Narayanan & Johnston, 2010). This segues to the more general question as to what physiological mechanisms exist, at the cellular and synaptic levels, that could actively regulate the balance of plasticity and stability through appropriate adjustment of the rules of activity‐induced synaptic alterations, thereby shaping the long‐term dynamics of modifiable synapses (Abraham, 2008).

Synaptic plasticity, as a potential neural substrate for learning and memory storage, is particularly well studied in the hippocampal formation (Malenka & Bear, 2004; Neves *et al*. 2008), and much about its molecular underpinnings has been learned from investigations at the excitatory CA3 to CA1 Schaffer collateral (SC) synapse, an integral component of the neural circuitry supporting spatial learning (Tsien *et al*. 1996; Heynen *et al*. 1996; Moser *et al*. 1998; Sheffield & Dombeck, 2019). These synapses are capable of undergoing bidirectional modification (both long‐term potentiation (LTP) and long‐term depression (LTD)), which relies primarily on the activation of postsynaptic *N*‐methyl‐d‐aspartate receptors (NMDARs) and the ensuing entry of calcium (Ca^2+^) into the dendritic spine (Malenka & Nicoll, 1993; Lynch *et al*., 1983; Malenka *et al*. 1988; Cummings *et al*. 1996). Given the biophysical requirements of both membrane depolarization and glutamate binding for their activation (Johnston & Wu, 1994), NMDARs are naturally poised to mediate input‐specific, Hebbian forms of synaptic learning (Magee & Johnston, 1997; Song *et al*. 2000). However, an account of Ca^2+^ signalling based solely around cell surface receptors is incomplete, as another source of Ca^2+^, the endoplasmic reticulum (ER) store, may be available to CA1 spines (Meldolesi & Pozzan, 1998). The ER, which extends into the dendritic processes of hippocampal neurons, has a heterogeneous distribution in individual spines (Spacek & Harris, 1997; Harris *et al*. 2015). Curiously, it occurs more often in larger spine heads: nearly 80% of the larger mushroom‐shaped spines have ER compared to about 20% of all spines in adult dendrites, as seen in serial EM reconstruction of rat CA1 pyramidal cell dendrites (Cooney *et al*. 2002).

Here, we consider a synapse‐specific form of metaplasticity due to Ca^2+^ stores associated with the ER in these large dendritic spines. This is in contrast to cell‐wide mechanisms which have been the focus of several previous studies. Our investigation is motivated by the observation that mature spines that have ER are associated with stronger synapses and have most likely been potentiated (Matsuzaki *et al*. 2001; Holbro *et al*. 2009; Sun *et al*. 2014; Chirillo *et al*. 2015). Further, recent imaging studies on cultured hippocampal neurons from mice reveal a dynamic picture of the ER distribution in spines (Toresson & Grant, 2005); ER can undergo rapid growth in individual spine heads on a time scale of minutes, which was shown to be regulated by NMDAR activation (Ng *et al*. 2014), and accompanies spine enlargement (Sala *et al*. 2001; Sala *et al*. 2005). This local remodelling of the ER in spines correlated with changes in spine morphology suggests an adaptive function for ER specifically at potentiated synapses, providing an interesting context to our investigation.

Several lines of previous experimental work indicate an involvement of the intracellular ER store in neuronal Ca^2+^ regulation (Verkhratsky, 2005), with possible implications for activity‐regulated plasticity processes (Mattson *et al*. 2000; Rose & Konnerth, 2001; Bardo *et al*. 2006). This still leaves open important questions regarding the role of stores in microdomain signalling in the context of its uneven distribution in CA1 spines. Given that small spines without ER are capable of undergoing potentiation, what additional functionality does ER introduce in the context of plasticity at individual synapses? Ca^2+^ release from ER is particularly associated with Ca^2+^ signalling underlying synaptic LTD (Malenka & Bear, 2004). Experimental studies on hippocampal/cortical LTD have implicated a requirement for group I metabotropic glutamate receptor (mGluR) signalling (particularly involving the mGluR5 subtype) in the induction of depression by prolonged low frequency synaptic stimulation (Kleppisch *et al*. 2001; Lüscher & Huber, 2010), and this was shown to depend on release of Ca^2+^ from inositol 1,4,5‐trisphosphate (IP_3_)‐sensitive intracellular stores (Stanton *et al*. 1991; Kato, 1993; Reyes & Stanton, 1996). These earlier findings were based on recordings of synaptic population responses; thus, pointed questions on the synapse specificity of mGluR signalling and Ca^2+^ store contribution could not be directly addressed. Two subsequent imaging‐based studies on plasticity at individual synapses provide more clarity in this regard. Glutamate uncaging at individual CA3–CA1 synapses in rat hippocampal slices (Holbro *et al*. 2009) was reported to evoke restricted Ca^2+^ release from ER in individual spine heads via IP_3_ signalling, and mGluR/store‐dependent depression induced by prolonged low frequency uncaging stimulation was found to be localized to synapses associated with such ER‐bearing spines. Results from a more recent similar study of spine structural plasticity (Oh *et al*. 2013) show that, at synapses associated with larger spines, the contribution of mGluR/IP_3_‐mediated store Ca^2+^ release in these spines is necessary for the induction of synaptic weakening with low frequency input trains. Together, the above findings suggest that, at least at a subset of synapses that are likely to have undergone experience‐dependent potentiation, IP_3_‐mediated Ca^2+^ release from spine ER may make a particularly significant contribution under weak stimulation, augmenting the NMDAR‐mediated Ca^2+^ influx to facilitate synaptic depression.

Computational models of hippocampal synapses typically attempt to account for experimental findings on LTP/LTD in terms of the postsynaptic Ca^2+^ elevation mediated by varying levels of NMDAR activation (Shouval *et al*. 2002; Graupner & Brunel, 2007; Rackham *et al*. 2010; Kumar & Mehta, 2011; Graupner & Brunel, 2012). These models provide a useful description of a canonical, or generic, synapse. Given that presence of spine ER may be restricted to a small proportion of synapses, the contribution of ER is likely to be ‘washed‐out’ and difficult to disambiguate based on coarse‐grained population readouts. Despite suggestive evidence for the contribution of internal stores to synaptic plasticity in area CA1, a clear description of their relevance to Ca^2+^ signalling in individual spines is lacking. To address this conspicuous gap in our understanding, we have undertaken a detailed characterization of the engagement of the ER Ca^2+^ store at an active CA3–CA1 synapse. Our modelling study of an ER‐bearing CA1 dendritic spine head integrates a detailed kinetic description of mGluR‐regulated Ca^2+^ release from ER with a realistic model for NMDAR Ca^2+^ signalling. Our analysis highlights the synapse‐level differences in Ca^2+^ signalling and plasticity arising from the presence of ER, and provides a novel perspective on the functional role of ER, as an intracellular Ca^2+^ store, in the postsynaptic context.

## Methods

**Figure 1:**
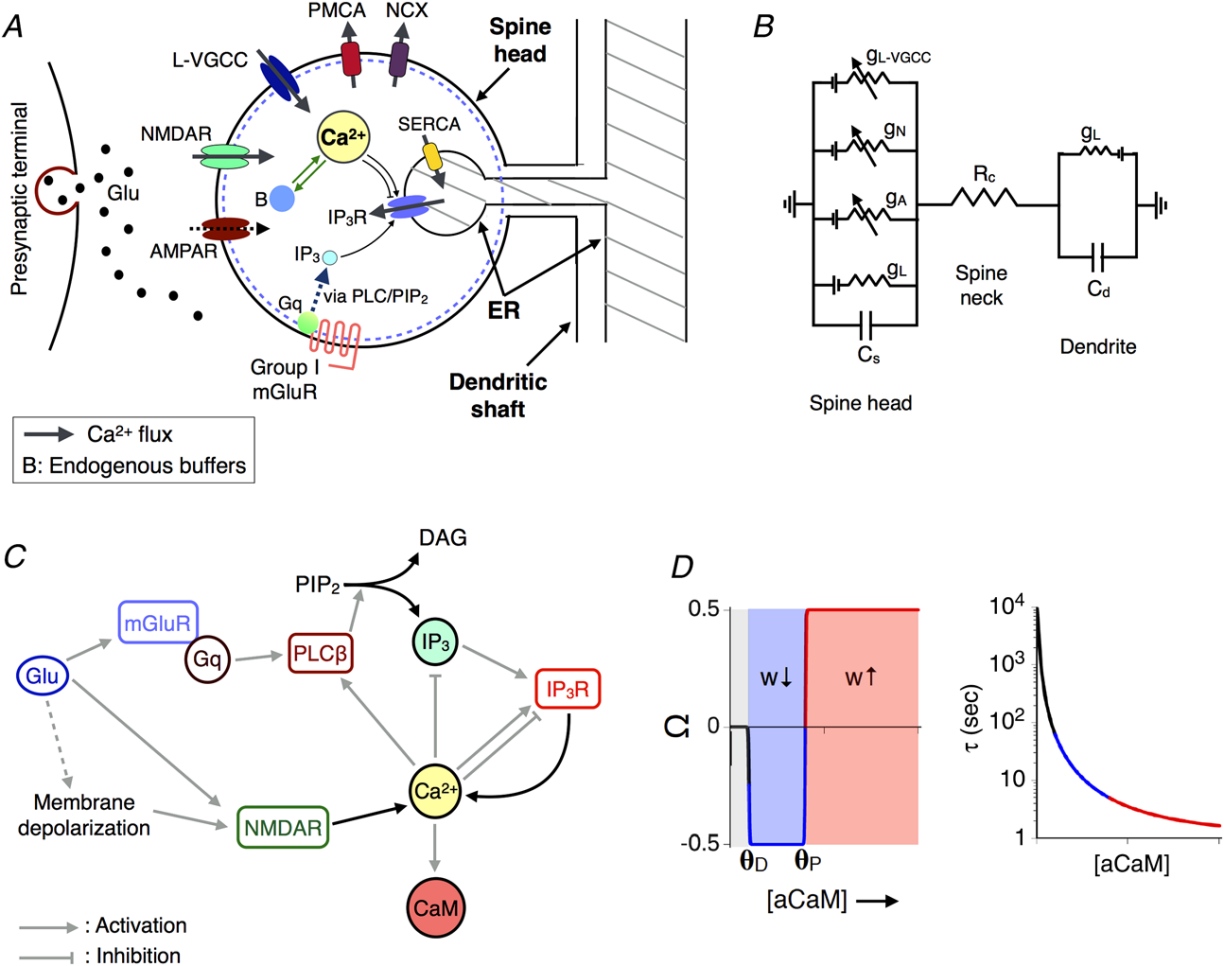
Modelling activity‐driven Ca^2+^ dynamics and plasticity at a dendritic spine with ER *A*, schematic representation of an ER^+^ CA1 spine head. Glutamate released into the synaptic cleft activates postsynaptic AMPA and NMDA receptors along with group I metabotropic receptors (mGluR), driving Ca^2+^ increase in the spine due to NMDAR‐gated entry from outside the cell and Ca^2+^ release from the IP_3_‐sensitive ER store. *B*, equivalent circuit diagram representing the spine head resistively coupled to a dendritic compartment by neck resistance *R*_C_. *C*, summary of the biochemical cascade involved in IP_3_ and Ca^2+^‐mediated Ca^2+^ release (ICCR) from ER. Ca^2+^‐activated calmodulin (aCaM) regulates synaptic plasticity at the spine. *D*, the Ca^2+^–CaM‐based synaptic plasticity model used in the present study. The functions Ω and τ are both functions of aCaM and together shape the dynamics of the synaptic weight (*w*). [Color figure can be viewed at wileyonlinelibrary.com]

We implemented a deterministic, single compartment model of a spine head on a hippocampal CA1 apical dendrite, and characterized the calcium (Ca^2+^) elevation and early long‐term plasticity driven by synaptic activation in the presence of an ER/spine apparatus (Fig. 1). The spine head was modelled as a sphere with fixed volume *V*_spine_ = 0.06 μm^3^, which approximates an average ER‐bearing spine head (ER^+^ spine) found experimentally (Holbro *et al*. 2009). While ER^+^ spines are on average larger than spines lacking an ER, our canonical spine head lies well within the reported dynamic range of spine sizes/synaptic strengths (Sabatini *et al*. 2002; Holbro *et al*. 2009), and thus may be considered potentially capable of undergoing plasticity in both directions (strengthening as well as weakening of synaptic efficacy). ER extending into spines is contiguous with the dendritic ER (Verkhratsky, 2005), and is typically found to occupy only a small fraction of the spine volume (≲5%) (Spacek & Harris, 1997); we assumed an ER‐to‐spine head volume ratio of 0.1. The spine head was assumed to be electrically coupled to its parent dendritic shaft by a thin ‘neck’, modelled as a passive electrical resistance, *R*_C_. Estimates for pyramidal cell dendrites suggest a coupling in the range of a few hundred megaohms (Yuste, 2013); here, we set *R*_C_ = 100 MΩ. Diffusive coupling of Ca^2+^ between the spine and dendrite was ignored, in accordance with Ca^2+^ measurements at mushroom spines (Sabatini *et al*. 2002; Bartol *et al*. 2015).

The various biophysical components comprising our model that collectively regulate the electrical and Ca^2+^ dynamics in the spine are described below. All model parameters and molecular concentrations used in our simulations are listed in Tables 1, 2, 3, 4.

**Table 1:** Parameters governing membrane voltage dynamics

Parameter	Symbol	Value
Avogadro number	*N*_a_	6.023 × 10^23^ mol^−1^
Electric charge per Ca^2+^ ion	*q*_Ca_	3.2 × 10^−19^ C
Volume of (spherical) ER^–^/ER^+^ spine head	*V*_spine_	0.06 μm^3^
Resting membrane potential in spine/dendrite	*u*_rest_	−70 mV
Resistive coupling between spine head and parent dendrite	*R*_C_	100 MΩ
AMPAR conductance parameter	*g*_A_	0.5 nS
AMPAR rise time constant	τ^r^_A_	0.2 ms
AMPAR decay time constant	τ^d^_A_	2 ms
AMPAR current reversal potential	*E*_A_	0 mV
NMDAR conductance parameter	*g*_N_	65 pS in model synapse (gives ∆Ca_EPSP_ = 0.2 μm)
NMDAR rise time constant	τ^r^_N_	5 ms
NMDAR decay time constant	τ^d^_N_	50 ms
NMDAR current reversal potential	*E*_N_	0 mV
VGCC activation offset	*u*_m_	−20 mV
VGCC activation slope	*k*_m_	5 mV
VGCC activation time constant	τ_m_	0.08 ms
VGCC inactivation offset	*u*_h_	−65 mV
VGCC inactivation slope	*k*_h_	−7 mV
VGCC inactivation time constant	τ_h_	300 ms
Maximum depolarization during BAP in dendritic compartment	*V*_0_	+67 mV
Fast time constant of BAP decay	τ_f_	3 ms
Slow time constant of BAP decay	τ_s_	40 ms
Effective number of co‐active synaptic inputs at dendritic compartment (rate‐dependent plasticity)	ρ_S_	5 × 10^5^ cm^−2^

**Table 2:** Reaction rate parameters for Ca^2+^ buffering and extrusion

Parameter	Symbol	Value
Kinetic rate constants for interaction between Ca^2+^ and calbindin (CB)	*k*_M0M1_ *k*_M1M2_ *k*_M1M0_ *k*_M2M1_ *k*_H0H1_ *k*_H1H2_ *k*_H1H0_ *k*_H2H1_	174 μm ^−1^ s^−1^ 87 μm ^−1^ s^−1^ 35.8 s^−1^ 71.6 s^−1^ 22 μm ^−1^ s^−1^ 11 μm ^−1^ s^−1^ 2.6 s^−1^ 5.2 s^−1^
Kinetic rate constants for interaction between Ca^2+^ and endogenous immobile buffer (CBP)	kf CBP kb CBP	247 μm ^−1^ s^−1^ 524 s^−1^
Kinetic rate constants for interaction between Ca^2+^ and endogenous slow buffer	kfs low kb slow	24.7 μm ^−1^ s^−1^ 52.4 s^−1^
Kinetic rate constants for interaction between Ca^2+^ and calmodulin (CaM)	*k*_C0C1_ *k*_C1C2_ *k*_C1C0_ *k*_C2C1_ *k*_N0N1_ *k*_N1N2_ *k*_N1N0_ *k*_N2N1_	6.8 μm ^−1^ s^−1^ 6.8 μm ^−1^ s^−1^ 68 s^−1^ 10 s^−1^ 108 μm ^−1^ s^−1^ 108 μm ^−1^ s^−1^ 4150 s^−1^ 800 s^−1^
Kinetic rate constants for PMCA pump	*k*_f_^PMCA^ *k*_b_^PMCA^ *k*_3_^PMCA^ *k*_L_^PMCA^	150 μm ^−1^ s^−1^ 15 s^−1^ 12 s^−1^ 3.33 s^−1^
Kinetic rate constants for NCX	*k*_f_^NCX^ *k*_b_^NCX^ *k*_3_^NCX^ *k*_L_^NCX^	300 μm ^−1^ s^−1^ 300 s^−1^ 600 s^−1^ 10 s^−1^

**Table 3:** **Reaction parameters for mGluR–IP_3_ signalling and ER Ca^2+^ handling (parameter values which have been changed from the original reference (** **Doi *et al*., 2005** **) are highlighted)**

Parameter	Symbol	Value
Time constant of glutamate pulse (alpha function)	τ_glu_	1 ms
Max. amplitude of glutamate pulse at mGluR location	*G*_max_	300 μm
Kinetic rate constants for mGluR5‐G protein activation	*a*_1f_ *a*_2f_ *a*_3f_ *a*_4f_ ***a*_1b_** ***a*_2b_** *a*_3b_ *a*_4b_ *a*_5_ *a*_6_ *a*_7_ *a*_8_	11.1 μm ^−1^ s^−1^ 11.1 μm ^−1^ s^−1^ 2 μm ^−1^ s^−1^ 2 μm ^−1^ s^−1^ **2 s^−1^** **2 s^−1^** 100 s^−1^ 100 s^−1^ 116 s^−1^ 0.001 s^−1^ 0.02 s^−1^ 6 s^−1^
Kinetic rate constants for phospholipase C (PLC) activation and production of IP_3_ and diacylglycerol (DAG)	*b*_1f_ *b*_2f_ *b*_3f_ *b*_4f_ *b*_5f_ *b*_1b_ *b*_2b_ *b*_3b_ *b*_4b_ *b*_5b_ *b*_6_ *b*_7_ *b*_8f_ *b*_9f_ *b*_8b_ *b*_9b_ *b*_10_ ***b*_11_** *b*_12_	300 μm ^−1^ s^−1^ 900 μm ^−1^ s^−1^ 800 μm ^−1^ s^−1^ 1200 μm ^−1^ s^−1^ 1200 μm ^−1^ s^−1^ 100 s^−1^ 30 s^−1^ 40 s^−1^ 6 s^−1^ 6 s^−1^ 2 s^−1^ 160 s^−1^ 1 μm ^−1^ s^−1^ 1 μm ^−1^ s^−1^ 170 s^−1^ 170 s^−1^ 8 s^−1^ **2 s^−1^** 8 s^−1^
Kinetic rate constants for IP_3_ degradation by IP_3_ 3‐kinase (IP3K)	*c*_1f_ *c*_1b_ *c*_2f_ *c*_2b_ *c*_3_	1111 μm ^−2^ s^−1^ 100 s^−1^ 100 μm ^−1^ s^−1^ 80 s^−1^ 20 s^−1^
Kinetic rate constants for IP_3_ degradation by IP_3_ 5‐phosphatase (IP3K)	*c*_4f_ *c*_4b_ *c*_5_	9 μm ^−1^ s^−1^ 72 s^−1^ 18 s^−1^
Parameters for IP_3_ receptor kinetics	*d*_1_ *d*_5_ *K*_inh_ *a*_2_ α	0.8 μm 0.3 μm 0.2 μm 2.7 μm ^−1^ s^−1^ 937.5 μm ^−1^ s^−1^ (corresponds to an open channel current of 0.15 pA when [Ca^2+^]_ER_ = 0.5 mm)
Parameters for Hill‐type SERCA kinetics	*K*_S_ *V*_S_	0.2 μm 1 μm s^−1^

**Table 4:** Concentrations of various chemical species in the model

Species	Value
Resting Ca^2+^ concentration in the spine cytosol ([Ca^2+^]_rest_)	50 nm
Resting IP_3_ concentration in the spine	0.1 μm
Ca^2+^ concentration in ER lumen (fixed)	250 μm
Fixed extracellular Ca^2+^ concentration ([Ca^2+^]_ext_)	2 mm
Total calbindin (CB) concentration	45 μm
Total concentration of endogenous immobile buffer (CBP)	80 μm
Total concentration of endogenous slow buffer	40 μm
Total calmodulin (CaM) concentration	50 μm
Surface density of PMCA pumps	1000 μm ^−2^
Surface density of NCX molecules	140 μm ^−2^
Total mGluR concentration in the spine	0.3 μm
Total PLC–PIP_2_ concentration in the spine	0.8 μm
Total PIP_2_ concentration	4 mm
Total Gq‐GDP concentration in the spine	1 μm
Total IP_3_ 3‐kinase (IP3K) in the spine	0.9 μm
Total IP_3_ 5‐phosphatase (IP5P) in the spine	1 μm
Number of IP_3_ receptors associated with spine ER (N_R_)	10–50

### Membrane voltage dynamics at the spine

The voltage at the postsynaptic membrane (*u*) is described by the following Hodgkin–Huxley (HH)‐type ordinary differential equation:

(1) A spine Cmdudt=−gLA spine u−u rest −IA−IN−IL- VGCC −u−udRC

It includes contributions from a passive leak current (*g*_L_), voltage‐dependent AMPA receptor (AMPAR)/NMDA receptor (NMDAR)‐gated currents (*I*_A_/*I*_N_) in the postsynaptic density (PSD), a high voltage‐activated L‐type Ca^2+^ current (*I*_L‐VGCC_), and passive electrical coupling to the dendritic shaft (*R*_C_) with *u*_d_ denoting the voltage of the dendritic compartment (Fig. 1 *B*). We assumed standard membrane parameters (capacitance *C*_m_ = 1 μF cm^−2^ and uniform leak conductance density *g*_L_ = 0.0002 S cm^−2^), and the resting membrane potential in both the spine and parent dendrite was set to *u*_rest_ = −70 mV (Graupner & Brunel, 2007).

AMPAR‐ and NMDAR‐gated currents were assumed to be transiently activated every time a synaptic input arrives, and both were modelled with linear *I–V* relations (reversal potentials *E*_A_, *E*_N_ = 0 mV) (Zador *et al*. 1990). The AMPAR conductance was modelled as the difference between two exponentials with a rise time constant of τAr=0.2 ms  and decay time constant τAd=2 ms  (Graupner & Brunel, 2007):

(2) g AMPAR t=gAe−t/τAd−e−t/τAr

for a glutamate pulse arriving at *t* = 0. The conductance parameter *g*_A_ was fixed at 0.5 nS across all our simulations (Zador *et al*. 1990). The glutamate dependence of the total NMDAR current was similarly modelled as a difference between exponentials with longer response times (τ^r^_N_ = 5 ms and τNd=50 ms ) (Narayanan & Johnston, 2010); the NMDAR conductance has an additional (multiplicative) dependence on the membrane potential, describing the Mg^2+^ block, which was modelled as a sigmoid function of the form *B*(*u*) = 1/(1 + 0.28 exp(−0.062*u*)) (Graupner & Brunel, 2007). The NMDAR conductance parameter *g*_N_ is a variable in our analysis, and adjusted to obtain different levels of spine Ca^2+^ elevation evoked by unitary synaptic input (see below). Trains of synaptic stimulation have been modelled as the sum of the above conductance waveforms (Zador *et al*. 1990).

The L‐type voltage‐gated Ca^2+^ current is regulated by a conductance of the form ĝ_L‐VGCC_(*t*) = *g*_L‐VGCC_ *m* ^2^_u_(*t*)*h*_u_(*t*), where the time dependence of the HH‐type activation and inactivation gating variables, *m*_u_(*t*) and *h*_u_(*t*), respectively, is governed by the following equations (Nowacki *et al*. 2011):

(3) $$\tau_{m}\frac{dm_{u}}{dt}=m_{\infty }\left(u\right)-m_{u}\;,\;\tau_{h}\frac{dh_{u}}{dt}=h_{\infty }\left(u\right)-h_{u}$$

where *m*_∞_(*u*) = 1/(1 + exp(−(*u* – *u*_m_)/*k*_m_)) and *h*_∞_(*u*) = 1/(1 + exp(−(*u* – *u*_h_)/*k*_h_)), with *u*_m,h_, *k*_m,h_ and τ_m,h_ listed in Table 1. The total (i.e., Ca^2+^) current through the L‐VGCC was described by a modified Goldman–Hodgkin–Katz (GHK) relation (De Schutter & Smolen, 1998) in order to correctly account for the large Ca^2+^ concentration gradient between the cytosol ([Ca^2+^]_rest_ = 50 nm) and the exterior of the cell ([Ca^2+^]_ext_ = 2 mm) under basal conditions. It is given by:

(4) IL− VGCC =0.078q Ca Namu2hugL− VGCC u× Ca −Ca ext e−0.078u1−e−0.078u

where *q*_Ca_ is the electric charge per Ca^2+^ ion, *N*_a_ is the Avogadro number, and the conductance parameter *g*_L‐VGCC_ is set according to the value of *g*_N_ such that the peak L‐VGCC Ca^2+^ influx rate during a backpropagating action potential (bAP) is comparable to that mediated by NMDAR in response to a glutamate pulse (i.e., during an evoked postsynaptic potential; EPSP), consistent with individual spine measurements (Sabatini *et al*. 2002).

### Calcium regulation

We assumed a basal steady‐state Ca^2+^ level of 50 nm in the spine head (Zador *et al*. 1990). The time course of the averaged, free (i.e. unbound) Ca^2+^ concentration during synaptic activity is described by the following equation:

(5) ddt Ca =JN+JL− VGCC +J ER +JB−J out

The terms *J*_ER_ represents the overall contribution of spine ER to Ca^2+^ activity (described in detail in the next two subsections), and was set to zero in the ER^−^ spine head, which in all other respects was identical to the ER^+^ spine. The Ca^2+^ influx through the NMDAR channel cluster, *J*_N_, was also described by a GHK‐type current term, and as in previous studies, we have assumed that the Ca^2+^ current constitutes 10% of the total NMDAR current (De Schutter & Smolen, 1998; Graupner & Brunel, 2007):

(6) JN=0.078gN Ca BuuV spine  Ca −Ca ext e−0.078u1−e−0.078uμM/s

where gN Ca =0.1 (g_N_/[Ca^2+^]_ext_)(*RT*/4*F* ^2^), with *R* denoting the universal gas constant (8.314 J mol^−1^ K^−1^), *F* = 96485 C mol^−1^ being the Faraday constant, and the temperature *T* set to 30°C.

**Figure 2:**
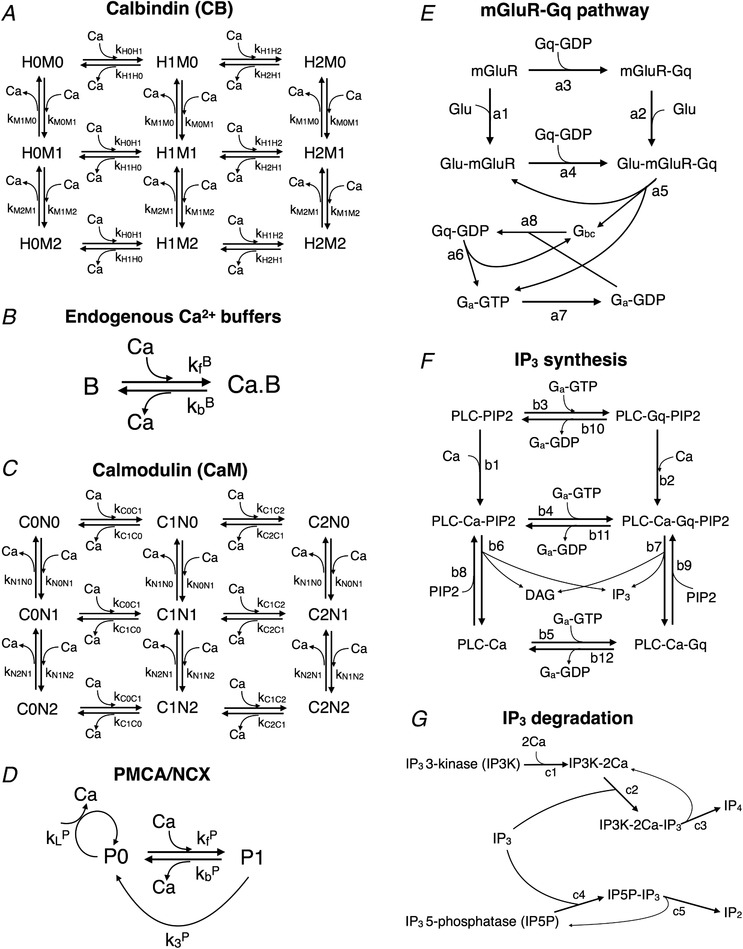
Reaction kinetics for various chemical species in the model *A*, nine‐state kinetic scheme for Ca^2+^ interaction with calbindin (CB). Each CB molecule possesses two medium affinity (M) and two high affinity (H) Ca^2+^ binding sites. *B*, first‐order reversible reaction describing the action of endogenous immobile and slow Ca^2+^ buffers. *C*, reaction network for the interaction of Ca^2+^ with the N and C lobes of calmodulin (CaM). *D*, Michaelis–Menten kinetic scheme to describe the action of plasma membrane Ca^2+^ pumps (P = PMCA or NCX). *E*, biochemical model for G‐protein (Gq) activation by group I metabotropic glutamate receptor (mGluR). *F*, detailed model describing the G‐protein‐mediated activation of PLC and subsequent production of IP_3_ and DAG via hydrolysis of PIP_2_. *G*, reaction steps involved in the enzymatic degradation of IP_3_ by IP_3_ 3‐kinase (IP3K), which has Ca^2+^‐dependent activity, and IP_3_ 5‐phosphatase (IP5P). (Note: several reaction steps in (*E–G*) are bidirectional (reversible), but have been represented as arrows in a single direction to avoid clutter. The full set of forward and backward kinetic rate constants is listed in Tables 2 and 3.)

The term *J*_B_ encapsulates the net effect of cytosolic buffers (Table 2). Calbindin D28‐k (CB) is a prominent fast‐binding mobile buffer in hippocampal neurons (Muller *et al*. 2005). The interaction of Ca^2+^ with CB is described by a detailed nine‐state kinetic scheme (Bartol *et al*. 2015) (Fig. 2 *A*). Each CB molecule carries two high‐affinity and two medium‐affinity binding sites for Ca^2+^. The total concentration of CB was taken to be 45 μm, as measured experimentally in CA1 pyramidal neurons (Muller *et al*. 2005). Besides CB, we included an endogenous ‘immobile’ Ca^2+^‐binding protein (CBP) with total concentration of 80 μm, as inferred previously from a spatial reaction–diffusion model of spine Ca^2+^ transients fit to single‐spine experimental data (Bartol *et al*. 2015). Its interaction with Ca^2+^ was described by a first‐order reversible reaction (Fig. 2 *B*). In addition, we included a slow buffer with a total concentration of 40 μm, the kinetic parameters for which were set 10 times slower than those for the immobile buffer (Naoki *et al*. 2005). We also included calmodulin (CaM) in our simulations. CaM is known to be present at a high concentration in CA1 dendritic spines (Faas *et al*. 2011). Due to the slower kinetics of its interaction with Ca^2+^ compared to CB, it has little impact during short Ca^2+^ transients (single synaptic events), but is expected to make a significant contribution to regulating free Ca^2+^ levels on longer time scales during persistent stimulation. We set the total CaM concentration to be 50 μm, which represents an average of several estimates found in the literature (Kakiuchi *et al*. 1982; Carafoli, 1987; Zador *et al*. 1990; Kubota & Waxham, 2010). We adopted a kinetic scheme used previously (Keller *et al*. 2008) to describe the reversible binding of CaM to Ca^2+^. Each CaM molecule comprises a high‐affinity C‐lobe and a low‐affinity N‐lobe, each of which can cooperatively bind up to two Ca^2+^ ions (Fig. 2 *C*).

In addition to the Ca^2+^ currents and buffers, the Ca^2+^ level in the cytosol is also regulated by efflux mechanisms operating at the plasma membrane (denoted by the *J*_out_ term in Eq. (5)), which set the time scale for the decay of Ca^2+^ transients in the spine head. We modelled efflux via plasma membrane Ca^2+^‐ATPase pumps (PMCA) and sodium–calcium exchangers (NCX), both of which were approximated by Michaelis–Menten‐like enzyme kinetics (Fig. 2 *D*). Surface densities and kinetic parameters for PMCA and NCX were adopted from a previous modelling study of CA1 spines (Bartol *et al*. 2015). In order to balance the extrusion of Ca^2+^ and ensure that Ca^2+^ in the cytosol can be maintained at a basal level of 50 nm in the absence of stimulation, the transporter equations also included leak terms (kL PMCA  and kL NCX ), which were set so as to exactly balance the efflux rate per pump molecule when the intracellular Ca^2+^ is held at 50 nm (Table 2).

### Model for IP_3_‐ and Ca^2+^‐induced calcium release (ICCR) from spine ER store

The intracellular Ca^2+^ store associated with spine ER/spine apparatus is a potential additional source of Ca^2+^ elevation in the spine head during synaptic activation. We modelled Ca^2+^ release from the ER pool evoked by the activation of G protein‐coupled group 1 metabotropic glutamate receptors (mGluR) (assumed to be present on the postsynaptic membrane), subsequent production of the second messenger IP_3_, and the downstream activation of IP_3_ receptors on the ER membrane (Fig. 2 *E–G*). We have adapted a previously published kinetic description of mGluR activation leading to phospholipase Cβ (PLCβ)‐mediated hydrolysis of phosphatidylinositol 4,5‐bisphosphate (PIP_2_) at cerebellar parallel fibre–Purkinje cell synapses (Doi *et al*. 2005), which itself borrows heavily from earlier models of metabotropic signalling proposed for generic mammalian neurons (Bhalla & Iyengar, 1999; Mishra & Bhalla, 2002). Glutamate uncaging at individual CA3–CA1 synapses (Holbro *et al*. 2009) indicates a delay of a few hundred milliseconds in Ca^2+^ release from the spine ER following the presentation of glutamate. A simpler (Hill function‐based) model for mGluR activation and IP_3_ turnover is unable to reproduce this experimental delay, prompting us to use the detailed kinetic description, which provides more flexibility in tuning the time course of IP_3_ production.

For the purpose of modelling mGluR–Gq activation, we simulated each glutamate pulse (synaptic input) as an α function with a time constant of τ_glu_ = 1 ms (glutamate is rapidly cleared from the synaptic cleft by the high density of glutamate transporters present on the surrounding astrocytic membrane; Bartol *et al*. 2015), and a peak concentration of 300 μm at the perisynaptically located mGluR (Doi *et al*. 2005) (scaled down from ∼1 mm at the PSD). We asked which of the parameters in the detailed mGluR–IP_3_ model most affected the latency in IP_3_ production. The IP_3_ time course was found to be robust to variation in all except a combination of three parameters (*a*_1b_, *a*_2b_ and *b*_11_), which were scaled down appropriately (*a*_1b_ and *a*_2b_ by a factor of 50 and *b*_11_ by a factor of 4) to introduce a few hundred milliseconds delay in the peak of the IP_3_ response following the initial input. All other parameter values governing Gq‐mediated PLC activation and IP_3_ turnover were taken to be the same as in the original study (Doi *et al*. 2005) (Table 3).

The gating of IP_3_ receptors was modelled with a reduced kinetic scheme adopted from a previous computational study of ER Ca^2+^ dynamics in neuroblastoma cells (Fink *et al*. 2000). IP_3_R activation was assumed to depend on the instantaneous levels of IP_3_ and Ca^2+^, modelled by the activation variables *m*_1_ = [IP_3_]/(*d*_1_ + [IP_3_]) and *m*_2_ = [Ca^2+^]/(*d*_5_ + [Ca^2+^]), with half‐activation constants *d*_1_ = 0.8 μm (lower affinity compared to *in vitro* measurements; Bezprozvanny *et al*. 1991) and *d*_5_ = 0.3 μm. IP_3_Rs are inactivated at higher calcium levels, which is accounted for by the slower kinetics of a third gating variable, *h*, given by the following equation:

(7) dhdt=a2K inh −K inh + Ca h

with the dissociation constant for inhibition, *K*_inh_, set equal to 0.2 μm. The Ca^2+^ influx through the IP_3_R‐gated channels is given by:

(8) J ICCR =αm1m2h3NRCa ER − Ca NaV spine μM/s

where *N*_R_ is the cluster size of the IP_3_R‐gated channels present in the spine head (estimated to be in the range of a few tens (Doi *et al*. 2005; Shuai *et al*. 2006; Ullah *et al*. 2012), and α sets the magnitude of the Ca^2+^ current through an open channel for unit concentration difference across the ER membrane. Single channel measurements suggest a type 3 IP_3_R open channel current of ∼0.15 pA at a Ca^2+^ concentration difference of 0.5 mm (Vais *et al*. 2010). The Ca^2+^ concentration in the ER lumen was assumed to remain constant in our simulations, and we used a value of [Ca^2+^]_ER_ = 250 μm throughout, which represents an average of several estimates in the literature (Meldolesi & Pozzan, 1998; Solovyova *et al*. 2002; LaFerla, 2002).

The ER can also contribute to cytosolic Ca^2+^ efflux through the sarco‐endoplasmic reticulum ATPase (SERCA) transporters present on the ER surface, which maintain the steep concentration gradient (∼10^3^–10^4^) across the ER membrane. The SERCA activity was assumed to have a Hill‐type dependence on the cytosol Ca^2+^ level (Fink *et al*. 2000), and is given by *F*_S_ = *V*_S_[Ca^2+^]^2^/([Ca^2+^]^2^ + *K*_S_^2^), with the half‐activation constant *K*_S_ set to 0.2 μm (Yasuda *et al*. 2004). As with the plasma membrane efflux pumps, we introduced a compensatory leak term at the ER surface, *k*_S_([Ca^2+^]_ER_ − [Ca^2+^]), where *k*_S_ is the SERCA leak rate, which balances the SERCA pump activity to help maintain a cytosolic [Ca^2+^] = 50 nm under resting conditions. The magnitude of maximum SERCA activity in the spine, *V*_S_, depends on the density of SERCA molecules on the ER membrane, ER surface area in the spine head and pump efficiency per molecule. As no estimate appropriate for a spine‐sized region was readily available, we obtained an approximate estimate for *V*_S_ by referring to previous experimental measurements on ER refilling rates in rat sensory neurons (Solovyova *et al*. 2002; Usachev *et al*. 2006). The refilling time constant there was found to be about 1–5 min. By making reasonable assumptions about the total cell volume, ER volume fraction, SERCA surface density (∼2000 per μm^2^; Means *et al*. 2006), and spine ER area (assumed to be 10% of the spine head area; Spacek & Harris, 1997), we arrived at an estimate of *V*_S_ = 1 μm s^−1^, which sets the scale for the contribution of SERCA activity to Ca^2+^ clearance in a typical ER^+^ spine head.

### Modelling synaptic activation

Unitary synaptic input at the spine head was modelled as a single pulse of glutamate, which transiently binds to postsynaptic mGluR, and concurrently activates AMPAR/NMDAR currents as described above to produce a small (∼few millivolts) depolarization of the postsynaptic membrane. The amplitude of spine Ca^2+^ evoked by NMDAR current during an EPSP (ΔCa_EPSP_) is considered to be in the range of a few hundred nM. For our control synapse (ER^−^ spine) we set the NMDAR conductance parameter *g*_N_ to 65 pS, which yields a ΔCa_EPSP_ = 0.2 μm, consistent with previous studies (Rackham *et al*. 2010; Kumar & Mehta, 2011). In order to assess the parameter dependence of our results, we also varied *g*_N_ to obtain a range of ΔCa_EPSP_ values (0.1–1 μm). The delay in Ca^2+^ release from the ER following the NMDAR‐mediated Ca^2+^ rise is measured with respect to the time of application of glutamate.

Induction of frequency‐dependent plasticity in ER^−^/ER^+^ spines was simulated with trains of regularly spaced synaptic inputs delivered over a range of different frequencies (0.1–20 Hz). In order to simulate the activation of Schaffer collateral (SC) fibres during the induction of LTP/LTD, we assumed that the local dendritic shaft (modelled as a passive compartment) receives synchronous input from multiple synapses (Zador *et al*. 1990; Schiegg *et al*. 1995), which amplifies the depolarization at the spine membrane through the passive resistive coupling (*R*_C_), leading to stronger activation of the NMDAR current at the spine head. The parameter ρ_S_, which sets the magnitude of the total number of co‐active synaptic inputs onto the dendritic compartment during SC stimulation, has been adjusted to evoke a depolarization of ∼15 mV at the spine head in response to a single input (Shouval *et al*. 2002). As the modest depolarization of the spine in this setting is insufficient for L‐VGCC activation, their contribution to the Ca^2+^ response was ignored for simplicity.

We also modelled the pairing of synaptic inputs with the strong depolarization of the postsynaptic membrane induced by backpropagating action potentials (bAPs) in the CA1 neuron, mimicking the conditions for the induction of spike timing‐dependent plasticity (STDP) (Wittenberg & Wang, 2006), which involves the activation of both the NMDAR and L‐VGCC. Dendritic bAPs were modelled as a voltage profile with a peak depolarization of *V*_0_ = 67 mV, and composed of a fast (τ_f_ = 3 ms) and a slow (τ_s_ = 40 ms) exponentially decaying components (Rackham *et al*. 2010; Kumar & Mehta, 2011):

(9) V bAP t=V0θt0.7e−t/τf+0.3e−t/τs

where θ(*t*) = 1 for *t* ≥ 0, and 0 otherwise, for a bAP arriving at *t* = 0. A bAP is simulated by feeding the above voltage profile to the dendritic compartment. During repetitive stimulation, each synaptic input is paired with either one or two bAPs (spike doublets/triplets). The timing difference between the pre‐ and postsynaptic firing, Δ*t*, is measured as the interval between the start of the glutamate pulse and peak of the bAP at the dendritic compartment. When a synaptic input is paired with a postsynaptic burst composed of two bAPs (spike triplet), Δ*t* is defined as the interval between the glutamate pulse and the peak of the *second* bAP; bAPs in a burst are separated by a fixed interval of 10 ms. By convention, glutamate release preceding the bAP is assigned a positive Δ*t*.

### Ca^2+^‐based plasticity model

Induction of long‐term modification at hippocampal/cortical synapses is governed by the magnitude and duration of local Ca^2+^ elevation at the dendritic spine (Malenka *et al*. 1992; Dudek & Bear, 1992; Yang *et al*. 1999). We adopted a previously proposed model for bidirectional plasticity (Shouval *et al*. 2002) to describe the induction of both rate and spike timing‐dependent synaptic efficacy changes at the ER‐bearing spine (Fig. 1 *D*). The model describes the dynamics of a (dimensionless) weight variable, *w* (a proxy for the postsynaptic AMPAR conductance), and its functional form approximates biophysically plausible descriptions of the regulation of AMPAR number and/or phosphorylation level by a combination of Ca^2+^‐activated kinases and phosphatases (Castellani *et al*. 2001, 2005). Following some earlier studies (Zador *et al*. 1990; Naoki *et al*. 2005; Faas *et al*. 2011), we modelled the dependence of *w* on the concentration of active calmodulin (aCaM) instead of the free Ca^2+^. Ca^2+^‐bound CaM is known to regulate the activation of several downstream effectors such as Ca^2+^/CaM‐dependent protein kinase II (CaMKII) (Pepke *et al*. 2010), PP2B/calcineurin (Kennedy, 2016) and protein kinase A (Chetkovich & Sweatt, 1993; Wang & Storm, 2003), which converge to mediate synaptic changes underlying early LTP/LTD expression (Barria *et al*. 1997; Derkach *et al*. 1999; Lee *et al*. 2000; Esteban *et al*. 2003). Although earlier models assumed that only the fully activated form of CaM (bound to 4 Ca^2+^ molecules) is relevant, recent studies indicate that even the partially bound forms are capable of regulatory activity (e.g. activation of CaMKII (Pepke *et al*. 2010)); thus, as a measure of CaM activity, we considered the total Ca^2+^‐bound CaM in the spine (aCaM). The dynamic range of aCaM is restricted by the availability of total CaM in the spine, unlike the Ca^2+^ response, which is not bounded and in principle can grow very large during persistent high‐frequency stimulation. The dynamical equation governing the Ca^2+^ dependence of *w* is given by

(10) τw aCaM dwdt=−w+Ωw aCaM

where the function Ω_w_ is modelled as the difference between two sigmoid functions (Shouval *et al*. 2002):

(11) Ωw aCaM =11+e−βP aCaM −θP−0.51+e−βD aCaM −θD

with slopes β_D_ = 60 μm ^−1^ and β_P_ = 60 μm ^−1^. The offsets θ_P_ and θ_D_ (θ_P_ > θ_D_) control the thresholds for the induction of LTP/LTD during synaptic stimulation: no plasticity is induced when aCaM levels remain below θ_D_, LTD is induced when aCaM is restricted by and large to the interval (θ_D_,θ_P_), and LTP induction requires higher levels of aCaM, exceeding the threshold θ_P_. The temporal factor τ_w_ is given by:

(12) τw aCaM =1+100.001+2 aCaM θD+θP2

and its parameters have been set in accordance with experimentally suggested rates for the induction of early LTP (∼seconds) and LTD (∼minutes) and the persistence of synaptic weight changes under resting conditions (∼1–3 h) (Shouval *et al*. 2002).

Rate‐dependent plasticity was simulated over the 0.1–20 Hz frequency range (in steps of 0.1 Hz) with 900 presynaptic SC inputs applied at each frequency, following earlier studies (Dudek & Bear, 1992; Shouval *et al*. 2002; Narayanan & Johnston, 2010). STDP induction was mimicked with a train of 100 pairings of pre/postsynaptic spiking presented at 5 Hz (Wittenberg & Wang, 2006), with the spike timing difference Δ*t* varied from −100 ms and 100 ms in steps of 1 ms. The weight variable *w* (initialized to 0 in all our simulations) integrates over the temporal spine Ca^2+^ signal evoked by these synaptic activation patterns, resulting in a net (cumulative) change Δ*w* at the end of the stimulation period. Spine ER was assumed to contribute to the Ca^2+^ pool in the spine driving changes in *w*, thereby modulating the induction of NMDAR‐dependent plasticity and giving rise to possible differences in Δ*w* relative to the reference ER^−^ spine. We characterized the nature of this differential effect of spine ER, ΔΔ*w*, as a function of various biophysical model parameters. The plasticity profile obtained in our model synapse is shaped by the choice of θ_P_/θ_D_ (assumed to be same for the ER^+^ and ER^−^ spines), which were appropriately set to have agreement with experimentally obtained plasticity curves. For the rate‐dependent plasticity, θ_P_ was adjusted such that LTP induction occurs for frequencies ≳15 Hz in the ER^−^ spine head (Dudek & Bear, 1992; Narayanan & Johnston, 2010) (although it represents an average obtained from a large number of synapses, we assigned this threshold to every individual synapse); we also examined the robustness of our results to variation in this parameter (*f*_P_ = 10–20 Hz). Different choices of the LTD threshold (θ_D_) for the ER^−^ spine have been similarly explored (*f*_D_ = 1–6 Hz). In the case of STDP, we referred to a previous experimental study (Wittenberg & Wang, 2006) which reports the average plasticity curve obtained from measurements at a population of CA3–CA1 synapses; here, again, we assigned the plasticity thresholds Δ*t*_D_/Δ*t*_P_ read off from the average curve to our reference synapse associated with an ER^−^ spine (implicitly assuming that only a minor proportion of the synapses recorded from are associated with an ER). We also followed this up by examining the sensitivity of the results to variation in Δ*t*_D_ and Δ*t*_P_ over ±10 ms windows.

All numerical simulations and analysis comprising this study were carried out in Python 2.7 using NumPy, SciPy and Matplotlib modules. Python code for running the model simulations is available at GitHub (https://github.com/gmcoderepo/ca1-spine-with-er).

## Results

### A calibrated kinetic model for mGluR–IP_3_ receptor signalling recapitulates salient features of experimental data on Ca^2+^ release from ER in dendritic spines

We considered an average CA1 pyramidal neuron dendritic spine head containing ER (an ER^+^ spine), described by a single‐compartment point model (Methods). The time course of spine Ca^2+^ evoked by synaptic activation is shaped by the coupled electrical and Ca^2+^ dynamics at the spine head, which involves contributions from various biophysical components, and passive electrical coupling with a dendritic compartment (Fig. 1 *A–C*). The spine ER store, modelled as an intracellular Ca^2+^ pool (fixed luminal concentration of 250 μm), contributes through IP_3_ receptor‐gated Ca^2+^ release (IP_3_‐ and Ca^2+^‐induced Ca^2+^ release; ICCR) and the uptake of cytosolic Ca^2+^ by SERCA pumps present on the ER membrane.

**Figure 3:**
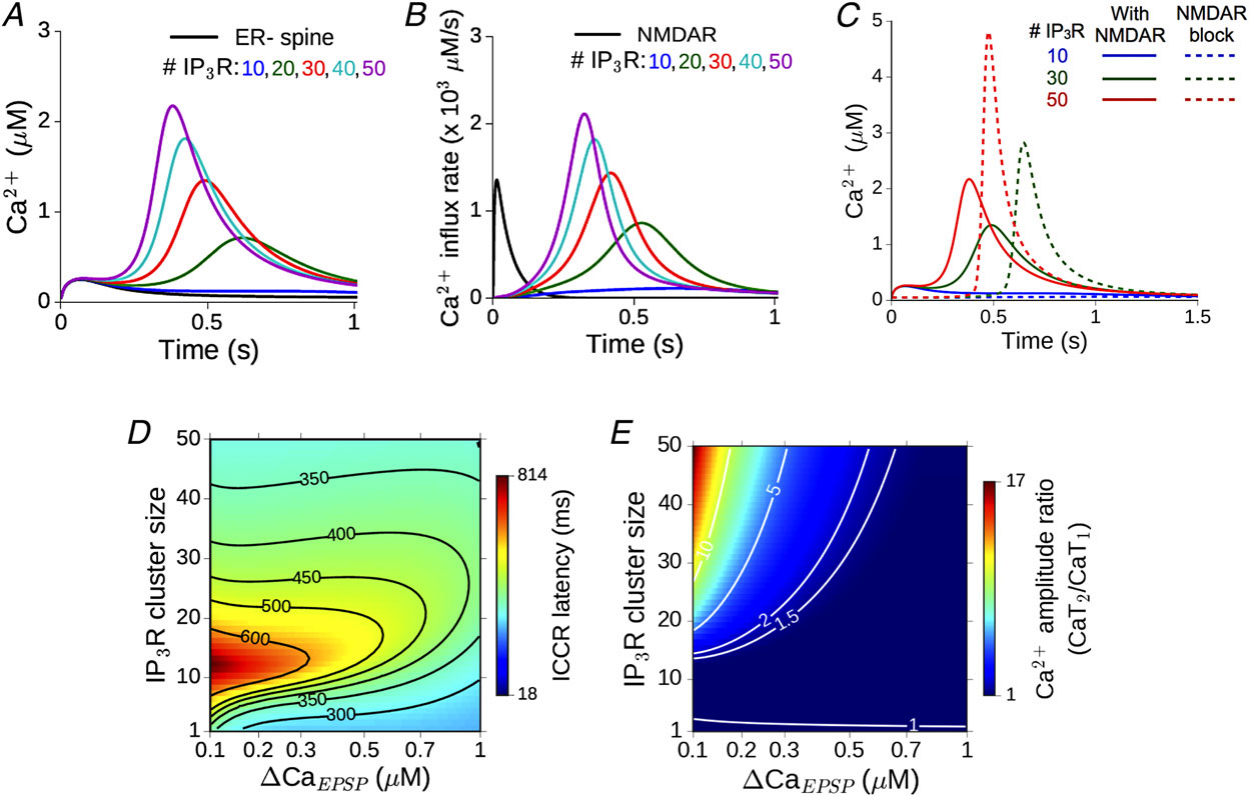
Glutamate evokes delayed release of Ca^2+^ from dendritic spine ER via mGluR signalling and IP_3_R activation *A*, response of the reference ER^−^ spine (black) to a single glutamate input is compared with the Ca^2+^ time course in ER^+^ spines with 10–50 IP_3_ receptors (coloured curves). The peak of the NMDAR‐mediated Ca^2+^ rise is ΔCa_EPSP_ = 0.2 μm. *B*, the underlying Ca^2+^ flux through NMDAR channels (black) and the delayed, temporally restricted flux through IP_3_R channels (coloured curves). *C*, comparison between delayed Ca^2+^ release from ER in the presence of NMDAR (continuous curves) and with NMDAR blocked (dashed curves) for different IP_3_R numbers. *D*, dependence of the delay in ICCR in the ER^+^ spine on the number of IP_3_ receptors and amplitude of the initial NMDAR‐evoked Ca^2+^ (ΔCa_EPSP_). The horizontal axis is linear in the NMDAR conductance parameter *g*_N_, but parameterized by Ca^2+^ for ease of interpretation; contours correspond to various values of the latency. *E*, dependence of ratio of the second to the first Ca^2+^ peak (illustrated in *A*) on the number of IP_3_ receptors and ΔCa_EPSP_. Contours shown for different values of the amplitude ratio. [Color figure can be viewed at wileyonlinelibrary.com]

In order to have a model of mGluR–IP_3_ signalling and ICCR that is appropriate for describing hippocampal synapses, we referred to Ca^2+^ imaging data from a previous experimental study of long‐term depression at individual excitatory CA3–CA1 connections (Holbro *et al*. 2009). Flash photolysis of caged glutamate was reported to evoke mGluR‐ and IP_3_‐dependent store Ca^2+^ release which was specific to ER^+^ spines, and trailed the initial NMDAR‐mediated spine Ca^2+^ transient (trial‐averaged delay of ∼470 ms). We adopted a detailed kinetic scheme (Doi *et al*. 2005) to describe the sequence of biochemical events linking the initial binding of glutamate to postsynaptic Gq‐coupled receptors (expressed on the extrajunctional membrane) to the eventual synthesis of IP_3_ and DAG via PIP_2_ hydrolysis mediated by activated PLCβ (Methods). We tuned the parameters regulating IP_3_ production rate in this model (Table 3) to reproduce the empirical estimate for the timing of the second Ca^2+^ peak relative to the arrival of glutamate (we chose parameters such that for a synapse with *N*_R_ = 30 IP_3_ receptors and ΔCa_EPSP_ ∼0.2 μm, the second Ca^2+^ peak occurs with a delay of *t* ≈ 480 ms). Figure 3 *A* and *B* shows the simulated Ca^2+^ response to application of a single pulse of glutamate at *t* = 0 in our model spine head with ER (coloured curves), which is compared with the reference ER^−^ spine (black curve). The latency as well as magnitude of Ca^2+^ release from ER is dependent on the number of IP_3_ receptors present. Although Ca^2+^ at low levels (≲0.3 μm) is a coagonist for IP_3_R activation (Finch *et al*. 1991), release of ER store Ca^2+^ in our calibrated model does not require the initial NMDAR‐mediated Ca^2+^ transient (Fig. 3 *C*), which, again, is consistent with experimental findings (Holbro *et al*. 2009).

The amplitude of the synaptically evoked Ca^2+^ (a direct readout for the NMDAR conductance) and the IP_3_ receptor cluster size directly determine the Ca^2+^ signal in the spine. Changes in the ICCR profile in our calibrated model corresponding to changes in these crucial components are summarized as heat maps in Fig. 3 *D* and *E*. These represent the dependence of the delay of ICCR (in ms) and the amplitude of the second Ca^2+^ peak relative to the first, respectively, over a biologically realistic range of parameter values. These parameter ranges yield a fairly broad distribution of possible outcomes, and for a small subset of parameter choices, the outcomes are consistent with the averaged experimental estimates (Holbro *et al*. 2009) (latencies of ∼400–500 ms and a second‐to‐first Ca^2+^ peak ratio of 1–5). Notably, the delay in synaptically evoked release of store Ca^2+^ has a non‐monotonic dependence on the IP_3_R cluster size: for a fixed ΔCa_EPSP_, the delay first increases, and then decreases with increasing number of IP_3_Rs. This dependence may be understood by noting that the IP_3_R cluster size sets the strength of the Ca^2+^‐dependent positive feedback loop driving IP_3_R activation in the presence of sufficient IP_3_. For higher IP_3_R numbers, this feedback can drive a self‐sustained burst of Ca^2+^ release from ER. The larger the cluster size, the more swiftly ICCR increases, while also speeding up the suppression of the slower Ca^2+^‐dependent inactivation variable *h*, which eventually shuts off the IP_3_R channel flux. Thus, the temporal profile of the ICCR transient is expected to advance (i.e. follow with a shorter lag) with increasing IP_3_R number. At small cluster sizes, on the other hand, the flux through IP_3_Rs is inadequate to drive a burst of ICCR even while IP_3_ is present. In this regime, there is no discernible second Ca^2+^ peak (e.g. the blue curve corresponding to 10 IP_3_Rs in Fig. 3 *A*), and the kinetics of the IP_3_R open fraction (i.e. the ICCR peak location) in this case is primarily shaped by the decaying NMDAR Ca^2+^ transient. The relatively minor contribution of ICCR only adds a small delay to this decay of the total spine Ca^2+^. This additional delay is proportional to the IP_3_R number, *N*_R_, thus accounting for the shift of the peak location of IP_3_R influx to longer latencies with increasing *N*_R_ in the regime of small IP_3_R numbers.

### mGluR‐mediated Ca^2+^ release from ER can facilitate the induction of synaptic depression with weak stimulation

**Figure 4:**
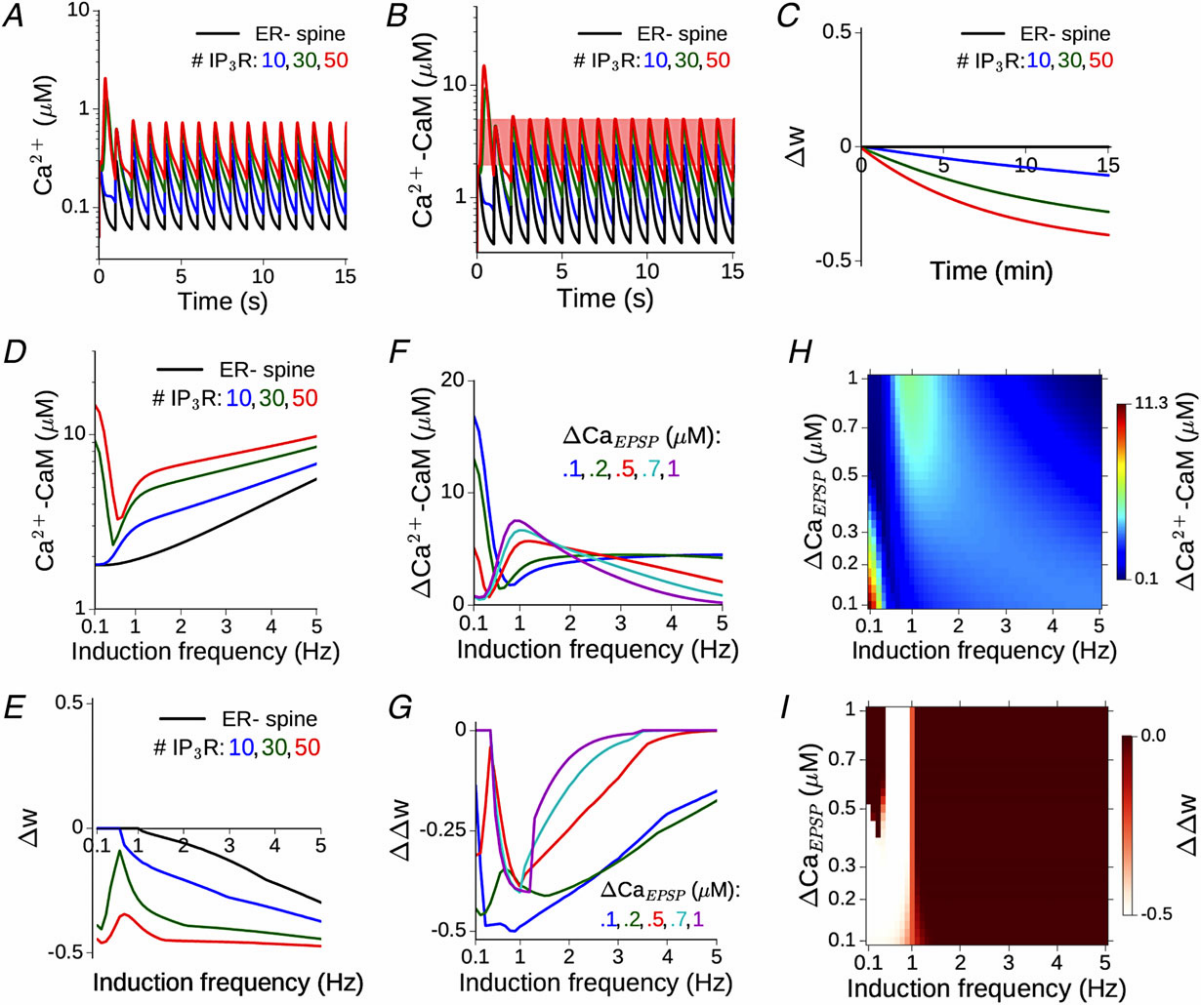
Ca^2+^ release from ER robustly enhances the spine Ca^2+^ signal and facilitates LTD induction at low input rates *A*, time course of Ca^2+^ evoked by repeated synaptic input at 1 Hz in the model ER^−^ spine (black) and an equivalent ER^+^ spine with 10–50 IP_3_ receptors. ΔCa_EPSP_ = 0.2 μM in the ER^−^ spine. *B*, the corresponding time dependence of Ca^2+^‐bound calmodulin (CaM) in the ER^−^/ER^+^ spine head. Total CaM concentration = 50 μM. *C*, change in the synaptic weight variable, *w*, driven by the activated CaM during a 1 Hz input train applied for 15 min (900 spikes). For this specific instantiation of the model, the plasticity threshold θ_D_ has been set to 2 μM, such that no LTD is induced in the absence of ER contribution, and LTD induction is facilitated with increasing flux through IP_3_Rs. *D*, the amplitude of CaM response to synaptic inputs in the 0.1–5 Hz range is compared between the ER^−^ spine (black) and equivalent ER^+^ spine with 10–50 IP_3_ receptors (coloured curves). *E*, change in the weight variable *w* at the end of 900 spikes in the ER^−^ (black) and ER^+^ spines (coloured curves), corresponding to *D*. θ_D_ is set to give no LTD in the ER^−^ spine for frequencies ≲ 1 Hz. *F*, the differential CaM response profile in an ER^+^ spine with 30 IP_3_Rs relative to the ER^−^ spine for different choices of ΔCa_EPSP_ (i.e. NMDAR conductance). *G*, differential LTD induction, ΔΔ*w*, in an ER^+^ spine with 30 IP_3_Rs relative to an ER^−^ spine for different choices of ΔCa_EPSP_. *H*, change in maximum CaM activation due to the presence of ER (30 IP_3_Rs) as a function of the NMDAR conductance and a range of low induction frequencies. *I*, difference in synaptic weight change between the ER^+^ and ER^−^ spine, as a function of the NMDAR conductance and induction frequency. Lighter colours indicate enhanced LTD response in the presence of ICCR. [Color figure can be viewed at wileyonlinelibrary.com]

We next simulated the calcium response in an ER^+^ spine head during the induction of synaptic weakening by low frequency afferent stimulation of the CA3–CA1 pathway. Figure 4 *A* shows an example of the Ca^2+^ time course evoked by a 1 Hz train of regularly spaced glutamate pulses. Binding of glutamate to postsynaptic AMPARs produces a small depolarization at the spine head (∼few millivolts), resulting in weak NMDAR activation and modest Ca^2+^ entry. Due to little overlap of successive Ca^2+^ events at low input rates, there is no build‐up of Ca^2+^ concentration in the spine over time. Figure 4 *A* compares the Ca^2+^ signal in the ER^−^ control spine (black curve) with the responses in the ER^+^ spine (coloured curves correspond to different numbers of IP_3_R). mGluR‐mediated Ca^2+^ release from spine ER contributes to the common pool of Ca^2+^ in the spine head and augments the NMDAR‐mediated Ca^2+^ signal.

In order to map the Ca^2+^ time course to plasticity, we followed the activation of calmodulin (CaM), which is included in our model and makes a significant contribution to the Ca^2+^ buffering capacity in the spine. Ca^2+^‐bound CaM is known to regulate a number of downstream signalling molecules that collectively determine changes in synaptic strength associated with early LTP/LTD; thus, it provides a suitable choice of input to the dynamical model governing the synaptic weight *w* (Methods and Fig. 1 *D*). The parameter θ_D_ in Eq. (11) decides the Ca^2+^–CaM threshold for LTD induction. For the example in Fig. 4 *B*, a range of θ_D_ choices is possible (indicated by the red band running parallel to the time axis) such that LTD can be induced at the ER^+^ spine, whereas the synaptic strength associated with the ER^−^ spine remains unaffected. This is illustrated in Fig. 4 *C* by a comparison between the time courses of *w*(*t*) in the ER^−^ and ER^+^ spines over 900 SC inputs for the specific choice of θ_D_ = 2 μm. The elevated Ca^2+^–CaM response in the ER^+^ spines (coloured curves) drives a slow induction of synaptic depression (Δ*w*<0) with repeated stimulation, which is absent in the ER^−^ spine (black curve). This particular instantiation of our model thus recapitulates experimental observations regarding the association of mGluR‐mediated store Ca^2+^ release with LTD induction at low stimulation rates (Reyes & Stanton, 1996; Oh *et al*. 2013).

In order to address the dependence of the results on the model parameters, we repeated our simulations across a range of synaptic input frequencies (0.1–5 Hz) and synaptically evoked spine Ca^2+^ amplitudes (ΔCa_EPSP_ = 0.1–1 μm). Figure 4 *D* shows the dependence of the maximum steady‐state amplitude of active CaM during persistent stimulation on the input frequency. The corresponding total change in the weight variable (Δ*w*) at the end of the stimulus train is shown in Fig. 4 *E* (*f*_D_ is fixed at 1 Hz). ICCR robustly enhances CaM activation to facilitate LTD induction over a range of low frequencies, although the contribution of ICCR is non‐monotonic in the input rate. This dependence may be accounted for by noting that the opening of IP_3_R is regulated by a combination of two factors: the Ca^2+^‐ and IP_3_‐dependent activation, and the level of inhibition (*h*). At low input rates (≲2 Hz), the IP_3_ level increases with the frequency of glutamate application. At the same time, the slowly changing *h* variable has less time to recover between successive inputs, the more frequently the inputs arrive; therefore, *h* decreases with increasing input frequency. The balance between these two competing factors (IP_3_‐mediated activation and inactivation mediated by *h*) shapes the overall profile of the IP_3_R open probability, and hence the IP_3_R Ca^2+^ flux, as a function of the frequency of glutamate input.

Figure 4 *F* and *G* compare the responses before and following spine ER acquisition over a range of low frequencies for different choices of the NMDAR conductance parameter (ΔCa_EPSP_), with a fixed number of IP_3_Rs (*N*_R_ = 30). The profiles of the *excess* CaM activation (Fig. 4 *F*) and differential plasticity outcome (Fig. 4 *G*) in the presence of spine ER depend on the amplitude of synaptically evoked Ca^2+^. However, in general, ICCR contribution enhances LTD induction over a range of low‐frequency inputs.

Elaborating on the above results, the heat maps in Fig. 4 *H* and *I* summarize the dependence of the maximal Ca^2+^–CaM response and plasticity output in the ER^+^ spine, relative to the ER^−^ reference spine, on the input frequency and NMDAR Ca^2+^ conductance. The vertical axes in both figures are linear in the NMDAR conductance parameter *g*_N_; however, to aid interpretation, they have been parametrized in terms of the NMDAR Ca^2+^ amplitude instead. As before, the frequency threshold for LTD induction is set to *f*_D_ = 1 Hz, and all results are for an IP_3_R cluster size of *N*_R_ = 30. ICCR is found to robustly enhance NMDAR‐driven LTD induction at lower frequencies (*f* ≲ 1 Hz). The underlying CaM activation in our model exhibits complex dependence on the input rate and ΔCa_EPSP_ (Fig. 4 *H*): the excess CaM response in the ER^+^ spine decreases with increasing ΔCa_EPSP_ at very low input frequencies (*f* ≲ 0.5 Hz), but this trend reverses at higher frequencies (*f* ∼ 1–2 Hz).

To see why this difference arises, we first considered the case of very low frequencies (*f* ≲ 0.5 Hz). Due to the delayed synthesis of IP_3_, the initial NMDAR Ca^2+^ elevation only contributes to ICCR by changing the slower inactivation variable *h* (and not through the activation variable *m*_1_), which decreases with increasing ΔCa_EPSP_. The level of *h* determines the magnitude of the subsequent ICCR (i.e. the amplitude of the second Ca^2+^ peak), and as *h* gets smaller with increasing ΔCa_EPSP_, so does the maximal IP_3_R Ca^2+^ flux (Fig. 4 *H*). At higher frequencies (∼1–2 Hz), on the other hand, there is insufficient time between one input and the next for IP_3_ to decay back to resting levels. As IP_3_ is now present at moderate levels when a glutamate input arrives, the NMDAR‐mediated ΔCa_EPSP_ switches its role: now it directly controls the activation of IP_3_Rs (through the instantaneous activation variable *m*_1_), while the slowly evolving *h*(*t*) only has a delayed effect on the IP_3_R open probability. Due to this switch in its role from inactivation to activation at higher input rates, an increase in *g*_N_ (i.e., ΔCa_EPSP_) is now associated with increased IP_3_R flux (Fig. 4 *H*). This explains the increase of ICCR with the NMDAR conductance. In sum, our results demonstrate that store Ca^2+^ contribution regulated by mGluR–IP_3_ signalling at a realistic synapse can robustly augment the NMDAR‐mediated Ca^2+^ response to weak synaptic stimulation, providing a basis to understand compromised hippocampal LTD associated with blocking of ER Ca^2+^ stores.

### The contribution of ICCR to spine Ca^2+^ dynamics is regulated by NMDAR activation and depends on the rate of synaptic stimulation

Our experimentally constrained model of mGluR–IP_3_ signalling indicates that spine ER can make a substantial contribution to the Ca^2+^ response evoked by weak synaptic inputs. Next, we examined the input frequency dependence of the ER contribution to spine Ca^2+^ dynamics during persistent synaptic activation. Higher input rates are associated with stronger NMDAR activation and greater build‐up of Ca^2+^ in the spine head, leading to a switch from LTD to LTP induction above some crossover frequency *f*_P_. We wished to address how the rate‐dependent bidirectional plasticity profile seen in the SC pathway (Dudek & Bear, 1992) is modulated by localized Ca^2+^ release from spine ER.

**Figure 5:**
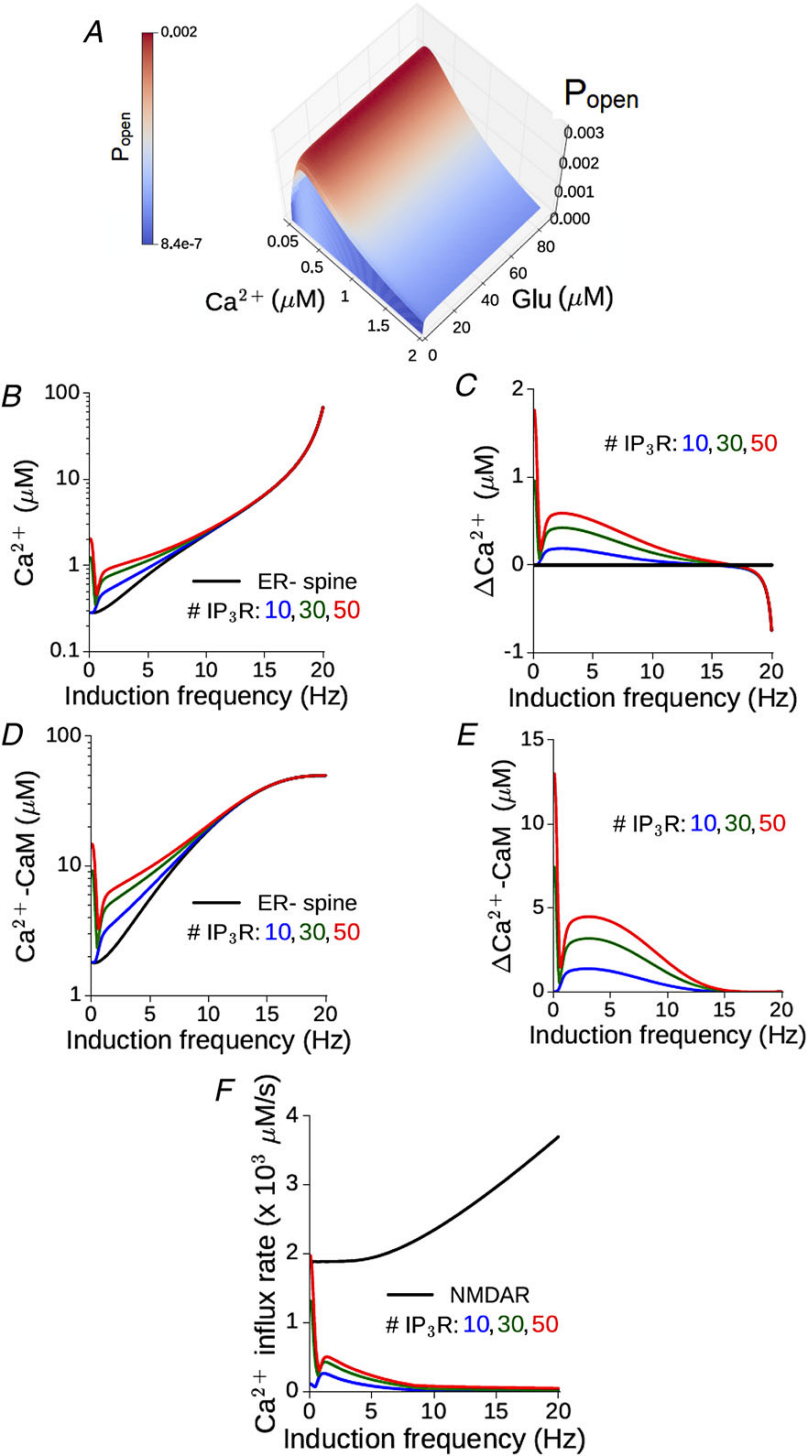
Augmented Ca^2+^–CaM response in a spine head in the presence of ER depends on the synaptic input rate and is suppressed at higher frequencies *A*, the steady state open probability of an IP_3_ receptor (*P*_open_) as a function of (constant) glutamate and Ca^2+^ concentrations. *B*, maximum Ca^2+^ level attained during persistent stimulation at different frequencies in the reference ER^−^ spine (black) and equivalent ER^+^ spine with different numbers of IP_3_Rs (coloured curves). *C*, non‐monotonic dependence of the differential Ca^2+^ responses in the ER^+^ spine on the input rate. *D* and *E*, the corresponding results for CaM activation as a function of the input rate. *F*, the maximum calcium influx rate through NMDA receptors (black) and different numbers of IP_3_ receptors (coloured curves) in an ER^+^ spine plotted against the input rate. (All results for the model synapse with peak NMDAR‐mediated Ca^2+^ response ΔCa_EPSP_ = 0.2 μM.) [Color figure can be viewed at wileyonlinelibrary.com]

Gating of IP_3_R in the spine can be regulated by the level of synaptic activation. This is summarized in Fig. 5 *A*, which shows the steady‐state open probability (*P*_open_) of the IP_3_R as a joint function of glutamate and spine Ca^2+^ concentration. Increasing the level of glutamate stimulation (e.g. in the context of rate‐based plasticity) has a direct effect on IP_3_ synthesis via mGluR–PLCβ activation. At the same time, it also drives increased postsynaptic NMDAR activation. The resulting elevation of spine Ca^2+^ level can affect both the production (via the mGluR pathway) and degradation (via the Ca^2+^ dependence of IP3K activity) of IP_3_, besides directly controlling *P*_open_ through the *m*_1_ and *h* variables (Methods). The broad strokes of this non‐linear dependence on glutamate and Ca^2+^ can be captured by the asymptotic steady‐state response of the IP_3_R over a range of (constant) glutamate and Ca^2+^ levels (Fig. 5 *A*). IP_3_R shows maximal activation for a Ca^2+^ concentration of ∼0.3 μm over a range of realistic glutamate levels, and is progressively inhibited with increasing Ca^2+^. Thus, in an ER^+^ spine, the contribution of ICCR is anticipated to diminish progressively with increasing NMDAR activation for a fixed glutamate signal (as in the case of STDP induction protocol). Given the weak dependence of *P*_open_ on the glutamate level as suggested by Fig. 5 *A*, we can also anticipate a similar graded contribution of ICCR with increasing NMDAR‐mediated Ca^2+^ elevation in the context of rate‐dependent plasticity. The interplay between NMDAR‐mediated Ca^2+^ entry, mGluR signalling and IP_3_R gating suggested by the above picture is characterized in detail in our synaptic model with realistic parameter settings.

We simulated a standard protocol consisting of repeated presynaptic stimulation (900 spikes) of the SC–CA1 pathway at different rates (0.1–20 Hz in steps of 0.1 Hz) (Methods). Figure 5 *B* compares the frequency–response profile of spine Ca^2+^ elevation (the maximum amplitude attained during the steady state) in the reference ER^−^ spine (black) with the ER^+^ spine for different numbers of IP_3_Rs (coloured curves). ICCR enhances the Ca^2+^ responses at lower frequencies, and its contribution steadily diminishes with increasing frequency above *f* ≈ 5 Hz; this is also highlighted by the profile of the excess Ca^2+^ amplitude in the ER^+^ spine relative to the ER^−^ spine, shown in Fig. 5 *C*. Similar dependence on the input rate is also observed for the activation of CaM in the ER^−^ and ER^+^ spines (Fig. 5 *D* and *E*). The dip in the differential Ca^2+^ signal below zero at higher frequencies (Fig. 5 *C*) is due to SERCA pump activity in the ER^+^ (but not the ER^−^) spine, which contributes to the extrusion of cytosolic Ca^2+^ and leads to a net lowering of the Ca^2+^ levels in the presence of ER. The action of SERCA Ca^2+^ efflux is revealed only at higher frequencies when ICCR in the spine is suppressed by the Ca^2+^‐dependent inactivation of IP_3_R. The inverse dependence of the ER Ca^2+^ contribution on the level of NMDAR activation is also highlighted by a direct comparison between the NMDAR and IP_3_R Ca^2+^ current profiles in the ER^+^ spine, which are shown in Fig. 5 *F*.

**Figure 6:**
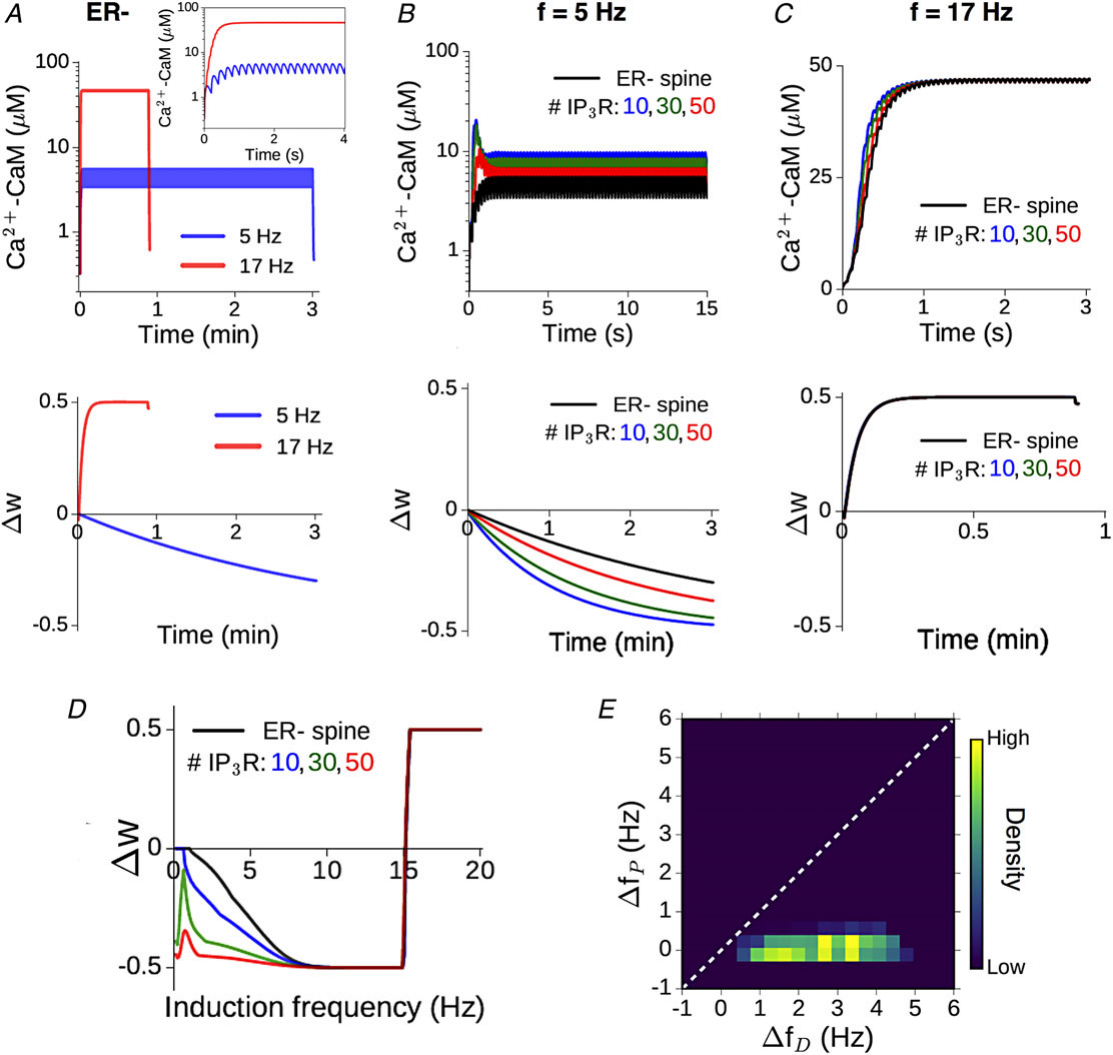
Graded contribution of ICCR to spine Ca^2+^ modulates the rate‐dependent bidirectional plasticity profile *A*, illustration of the CaM response (top) and plasticity induction (bottom) at the reference ER^−^ spine for 900 presynaptic spikes applied at two different rates, 5 (blue) and 17 (red) Hz. Inset shows a magnified view of the first 4 s of the Ca^2+^–CaM time course. Plasticity parameters have been adjusted to yield LTD and LTP thresholds *f*_D_ = 1 Hz and *f*_P_ = 15 Hz, respectively. *B*, enhancement of NMDAR‐mediated CaM activation (top) and LTD induction (bottom) at 5 Hz stimulation due to ER Ca^2+^ contribution for different IP_3_R cluster sizes. *C*, time course of CaM activation (top) and potentiation (bottom) at 17 Hz stimulation in the ER^−^ spine (black) and ER^+^ spine for different IP_3_R cluster sizes (coloured curves). Due to the suppression of ICCR at higher frequencies, the presence of ER makes little difference in this case. In *B* and *C*, only the initial phase of the (much longer) Ca^2+^–CaM time course is shown for clarity. *D*, dependence of the total weight change (Δ*w*) induced by prolonged stimulation (900 spikes) on the synaptic input rate at the ER^−^ spine (black) and corresponding ER^+^ spine for different IP_3_R numbers (coloured curves); *f*_D_ = 1 Hz and *f*_P_ = 15 Hz. Plasticity is enhanced in the ER^+^ spine at low frequencies, with diminished modulation at higher (LTP‐inducing) rates. *E*, graded contribution of ICCR to the modulation of plasticity is quantified in terms of the relative shifts in the LTD/LTP thresholds in the ER^+^ spine (relative to the ER^−^ control spine). The heat map is a distribution of Δ*f*_D_/Δ*f*_P_ values obtained by random sampling (5000 times) of the plasticity thresholds *f*_D_ and *f*_P_ (over 1–6 Hz and 10–20 Hz, respectively), and IP_3_R number (10–50). (All results for a synapse with NMDAR‐mediated ΔCa_EPSP_ = 0.2 μM.) [Color figure can be viewed at wileyonlinelibrary.com]

Spine Ca^2+^ elevation drives a change in the synaptic weight variable *w* (Eq. (10)). This is illustrated for the ER^−^ (control) spine in Fig. 6 *A*, which shows the temporal profile of Ca^2+^–CaM (Fig. 4 *A*, top) and the corresponding evolution of the *w* variable (Fig. 6 *A*, bottom) over 900 spikes at two different input rates, 5 and 17 Hz, associated with LTD and LTP, respectively. (The plasticity thresholds have been adjusted to have *f*_D_ = 1 Hz and *f*_P_ = 15 Hz at the ER^−^ spine.) The differential effect of ER is displayed separately for the 5 Hz (Fig. 6 *B*) and 17 Hz (Fig. 6 *C*) examples. At 5 Hz, ICCR makes an appreciable contribution to the spine Ca^2+^ elevation, and thus to the rate and amplitude of the resulting synaptic changes, with a larger number of IP_3_Rs associated with stronger synaptic depression (Fig. 6 *B*, bottom). In contrast, at 17 Hz, due to the strong suppression of ICCR by the NMDAR‐driven persistent Ca^2+^ elevation, there is little difference in the response in the ER^−^ and ER^+^ spines (Fig. 6 *C*), resulting in nearly the same plasticity outcome (strong potentiation) at the end of the stimulation (Fig. 6 *C*, bottom).

We quantified the induced plasticity profile across the full range of synaptic input rates (0.1–20 Hz), which is shown in Fig. 6 *D*. The total synaptic weight change (Δ*w*) at the end of the stimulus train is plotted as a function of the input rate *f* for the reference ER^−^ spine (black) and following ER acquisition for different numbers of IP_3_Rs (coloured curves). Consistent with our expectation from Fig. 5 *A*, we found that ICCR enhances plasticity at lower input frequencies, leading to a broadening of the effective LTD window. Due to the Ca^2+^‐dependent suppression of ICCR at higher frequencies, the profiles of Δ*w* for synapses associated with the ER^−^ and ER^+^ spines are near‐identical above *f* ≈ 10 Hz.

The differential modulation of LTD and LTP suggested by the above results for our model synapse (ΔCa_EPSP_ = 0.2 μm) is characterized in terms of the relative broadening of the depression and potentiation windows. A simple way to capture the overall modulation of the plasticity curve is by estimating changes in the threshold frequency for LTD induction (Δ*f*_D_) and LTP induction (Δ*f*_P_) with the inclusion of ER, relative to the ER^−^ spine. The plasticity thresholds for the ER^−^ spine (*f*_D_ and *f*_P_) were repeatedly sampled at random from 1–6 Hz for *f*_D_ and 10–20 Hz for *f*_P_ to account for experimental uncertainties in these estimates, as well as to as assess the variability in the model output. The resulting distribution of (Δ*f*_D_, Δ*f*_P_) values (aggregate of 5000 runs over 10–50 IP_3_Rs) is visualized as a heat map in Fig. 6 *E*, and on the whole, it suggests selective enhancement of LTD induction in the presence of ER. In summary, our analysis of the model spine head suggests a graded, frequency‐dependent contribution of ER to spine Ca^2+^ signalling and plasticity, with steadily diminishing contribution of ICCR at higher input frequencies.

### Ca^2+^ release from the IP_3_‐sensitive ER store selectively enhances synaptic depression during spike timing‐dependent plasticity

We next examined the involvement of ER in spine Ca^2+^ dynamics during trains of pre‐ and postsynaptic action potentials (APs), which simulate the conditions for induction of timing‐ dependent synaptic plasticity (Markram *et al*. 1997; Nishiyama *et al*. 2000). In the context of hippocampal CA3–CA1 synapses, pairing of every presynaptic spike with one bAP at theta frequency (5 Hz) was found to induce only synaptic weakening, and LTP induction requires repeated pairing of glutamate release with AP bursts (two bAPs) in the postsynaptic neuron (Wittenberg & Wang, 2006; Tigaret *et al*. 2016). We made use of these experimental data on STDP, specific to the CA3–CA1 synapse, to constrain our model for spine Ca^2+^ signalling, and examined the contribution of ICCR during this form of plasticity.

**Figure 7:**
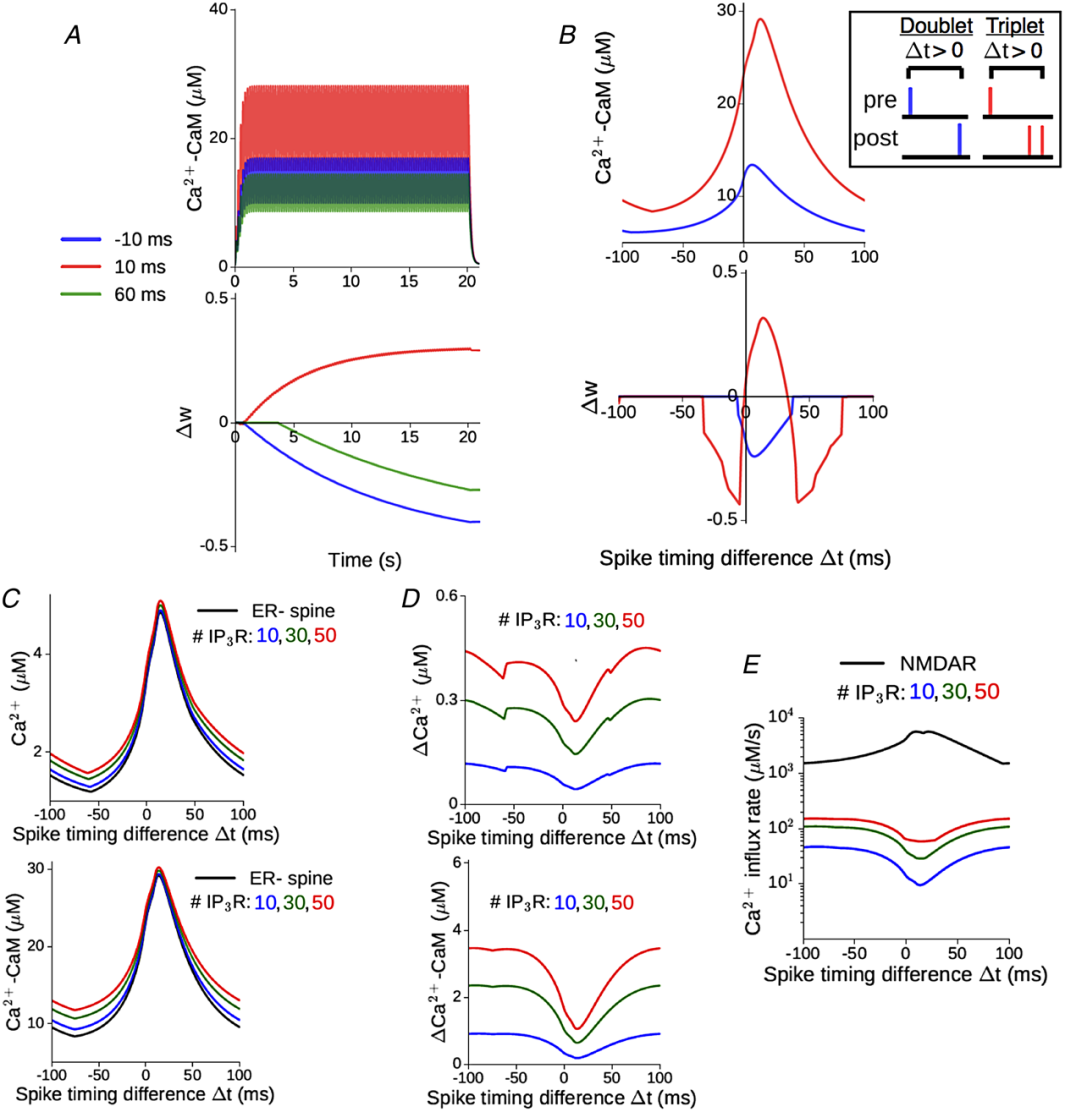
Differential Ca^2+^–CaM response in the presence of spine ER depends on NMDAR activation regulated by the spike timing difference *A*, illustration of the time course of CaM activation (top) and plasticity induction (bottom) at the reference ER^−^ spine in response to 100 spike triplets (1 EPSP + 2 bAP) at 5 Hz, for three different spike timing differences (Δ*t*): −10, +10 and +60 ms. Plasticity parameters have been chosen so as to yield depression at −10 and +60 ms, and potentiation at +10 ms, consistent with previous measurements (Wittenberg & Wang, 2006). *B*, top, maximum CaM activation attained during prolonged paired stimulation as a function of Δ*t*, when every synaptic input is paired with either 1 (blue) or 2 (red) bAPs. The inset shows details about the STDP stimulus pattern (doublet *vs*. triplet pairing, and convention for positive Δ*t*). Bottom, total weight change at the end of the stimulation, plotted as a function of Δ*t* for the doublet (blue) and triplet (red) spike pairings. No potentiation is induced in the former case. Parameters same as in *A*, in order to have overall consistency with experimental profiles. *C*, the amplitude of Ca^2+^ (top) and CaM activation (bottom) in the ER^−^ spine (black) *vs*. ER^+^ spine for different IP_3_R numbers (coloured curves), during presentation of 100 spike triplet pairings at 5 Hz over a range of Δ*t*. *D*, the corresponding excess Ca^2+^ (top) and CaM (bottom) responses in the ER^+^ spine relative to the ER^−^ control. *E*, maximum rate of Ca^2+^ influx into the ER^+^ spine cytosol through NMDAR channels (black) and different numbers of IP_3_Rs during paired stimulation over a range of Δ*t* values. (All results for a synapse with ΔCa_EPSP_ = 0.2 μM.) [Color figure can be viewed at wileyonlinelibrary.com]

We simulated the Ca^2+^ dynamics in our synaptic model during a sequence of pre‐ and postsynaptic spikes. Every glutamate input is separately paired with either one bAP (spike doublets) or two bAPs (spike triplets), and these paired stimuli are presented 100 times at a fixed rate of 5 Hz (Methods). Figure 7 *A* illustrates the time course of Ca^2+^–CaM (top) and the corresponding change in the synaptic weight (bottom) in the ER^−^ (control) spine head during the triplet stimulation, for three different choices of the pre‐post spike timing difference, Δ*t*. By appropriately adjusting the plasticity thresholds Δ_D_ and Δ_P_, our ER^−^ spine model can show broad agreement with the experimentally reported plasticity profiles. Figure 7 *B* (top) shows the dependence of the Ca^2+^–CaM amplitude on the spike timing difference (Δ*t*) in the doublet (blue) and triplet (red) cases. The temporal proximity and ordering of pre‐ and postsynaptic inputs, together, control the NMDAR activation level, which in turn decides the maximum Ca^2+^ elevation in the spine during persistent stimulation. The corresponding synaptic weight changes (Δ*w*) induced by the STDP inputs are displayed in Fig. 7 *B* (bottom). Consistent with experimental data, the doublet protocol only induces synaptic weakening over a restricted range of Δ*t* values (Fig. 7 *B*, bottom, blue curve). On the other hand, the triplet protocol induces potentiation over an ∼35 ms window of positive Δ*t* values, flanked by two ∼40 ms windows of depression, one for Δ*t* < 0 and a second for causal pre/post pairings with longer time differences (Δ*t* ≥ +35 ms) (Fig. 7 *B*, bottom, red curve).

How does ER modulate spine Ca^2+^ signalling during STDP induction? Figure 7 *C–E* compares the activity‐driven responses of an ER^+^ spine (with different IP_3_R cluster sizes) with the ER^−^ reference spine, as a function of the spike timing difference. Release of ER store Ca^2+^ augments both the Ca^2+^ and Ca^2+^–CaM in the spine head (Fig. 7 *C*), and this excess response in the ER^+^ spine relative to the ER^−^ spine indirectly depends on the spike timing difference, which regulates the NMDAR‐mediated Ca^2+^ entry into the spine (Fig. 7 *D*). This inverse dependence of the ICCR contribution on NMDAR activation level is reflected in the NMDAR and IP_3_R Ca^2+^ current profiles, shown in Fig. 7 *E*. Stronger NMDAR activation, particularly at small positive spike timing differences (0 < Δ*t* ≤ +40 ms), is associated with reduced ICCR, which follows from the Ca^2+^‐dependent inhibition of IP_3_ receptors at sustained Ca^2+^ levels above ∼0.3 μm. These trends broadly agree with the results from our previous simulations of rate‐based plasticity (Fig. 5).

**Figure 8:**
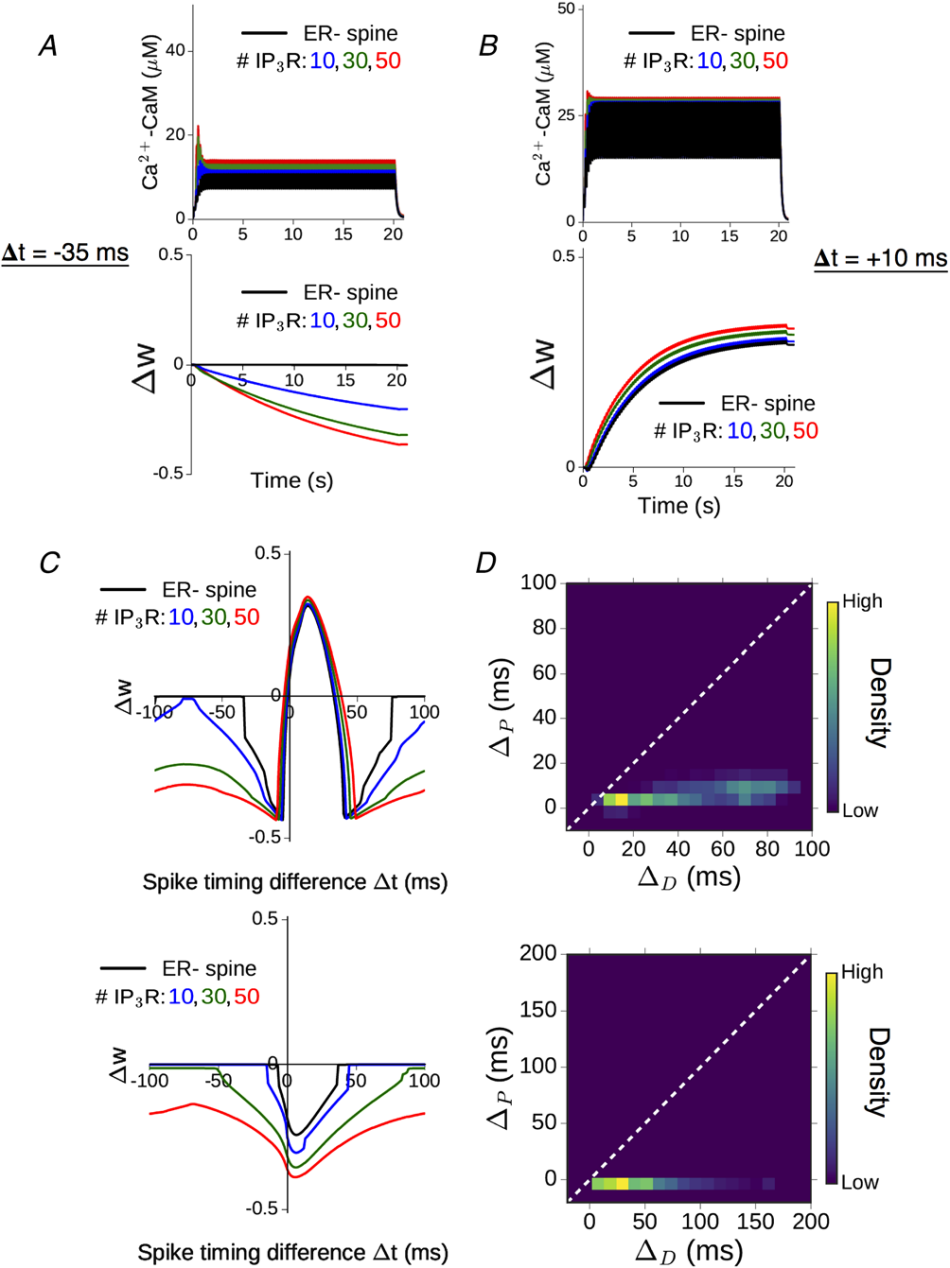
NMDAR‐dependent contribution of ICCR to spine Ca^2+^ response modulates the STDP profiles *A*, time course of the spine CaM activation (top) and plasticity variable Δ*w* (bottom) in response to triplet pairing stimulation with Δ*t* = −35 ms. Results shown for the ER^−^ control spine (black) and ER^+^ spine with different IP_3_R numbers (coloured curves). *B*, results obtained for Δ*t* = +10 ms. (Parameters here are same as in Fig. 5, with no plasticity induced in the ER^−^ spine for Δ*t* = −35 ms.) *C*, comparison of the plasticity profiles (total weight change Δ*w*) obtained in the absence of ER (black) and with different levels of ICCR (coloured curves) in response to 100 pairings of spike triplets (top) and doublets (bottom). *D*, quantification of relative changes in LTD and LTP window widths when ICCR is present, for the triplet (top) and doublet (bottom) stimulation patterns. Heat maps represent distributions of (Δ_D_, Δ_P_) values obtained from random sampling (5000 times) of the STDP thresholds Δ*t*_D_ and Δ*t*_P_ in the ER^−^ control spine from 20 ms windows centred on −35 and +35 ms, respectively, and with 10–50 IP_3_Rs. [Color figure can be viewed at wileyonlinelibrary.com]

Transient elevation of Ca^2+^–CaM levels drives the induction of plasticity, governed by Eq. (11). The temporal profiles of activated CaM and corresponding plasticity outcomes in the ER^−^/ER^+^ spines are illustrated for two representative spike timing differences in Fig. 8 *A* and *B*. For Δt = −35 ms, the amplitude of active CaM in the ER^−^ spine lies just below the threshold for the induction of synaptic depression (Fig. 8 *A*, black curves). The additional release of ER store Ca^2+^ increases the total CaM activation above the LTD threshold, inducing strong synaptic weakening (Δ*w* < 0) in the presence of ER (Fig. 8 *A*, coloured curves). When Δ*t* = +10 ms, ICCR makes a relatively modest contribution to the total Ca^2+^ response in the spine head (Fig. 8 *B*, top), and this yields a small net enhancement of synaptic strengthening compared to the ER^−^ spine (Fig. 8 *B*, bottom). We simulated STDP inputs to our synaptic model over the full range of allowed spike timing differences (−100 ms ≤ Δ*t* ≤ +100 ms), and the plasticity profiles obtained for the triplet and doublet protocols are shown in Fig. 8 *C*. Ca^2+^ release from spine ER modulates the overall STDP curve for triplet inputs (Fig. 8 *C*, top), and synaptic weakening is elicited over a broader range of spike timing differences compared to the ER^−^ spine. Consistent with the reduced contribution of ICCR at small positive Δ*t* (Fig. 7 *D* and *E*), presence of ER introduces relatively less broadening of the LTP induction window. In the case of doublet inputs (Fig. 8 *C*, bottom), ICCR extends the window for induction of synaptic depression over a broader range of spike timing differences relative to the ER^−^ spine, the extent of which scales with the number of IP_3_R present.

The differential modulation of the depression and potentiation windows seen in the above data was quantified in terms of the relative change in widths of the LTD and LTP windows (Δ_D_ and Δ_P_) in the presence of ER. Repeated random sampling of the STDP thresholds Δ*t*_D_/Δ*t*_P_ for the ER^−^ spine (from ±10 ms windows centred on Δ*t*_D_ = −35 ms and Δ*t*_P_ = +35 ms) yields a distribution of possible (Δ_D_, Δ_P_) pairs. The results (aggregate of 5000 samples, 10–50 IP_3_Rs) are represented by separate heat maps for the triplet and doublet input patterns (Fig. 8 *D*). The overall distribution, which, by and large, is confined to the lower triangle (Δ_D_ > Δ_P_), indicates that the contribution of ICCR can potentially extend the LTD window by several tens of milliseconds, whereas its effect on the LTP window is comparatively less. Taken together, Fig. 8 *C* and *D* demonstrates the relative broadening of the LTD window in the presence of ICCR, which is fairly robust to variation of the plasticity thresholds and the IP_3_R cluster size. In summary, we found that mGluR‐mediated Ca^2+^ release from spine ER during correlated activation of pre‐ and postsynaptic neurons promotes the induction of synaptic depression over a broader range of temporal activation patterns (Δ*t*), with relatively less influence on LTP induction.

### Differential contribution of ICCR to LTD and LTP induction is a general consequence of IP_3_ receptor kinetics

The foregoing analysis of our detailed synaptic model highlights the potential contribution of ICCR to activity‐driven Ca^2+^ signalling in the spine, and its regulation by NMDAR‐mediated Ca^2+^ entry. These results were obtained with a specific choice of the NMDAR conductance, a key parameter controlling the dynamic range of NMDAR‐mediated Ca^2+^ responses in the spine. To assess the robustness/sensitivity of our model predictions, we repeated our simulations by varying the conductance parameter *g*_N_ so as to span a physiologically plausible range of synaptically evoked Ca^2+^ amplitudes (ΔCa_EPSP_ = 0.1–1 μm).

**Figure 9:**
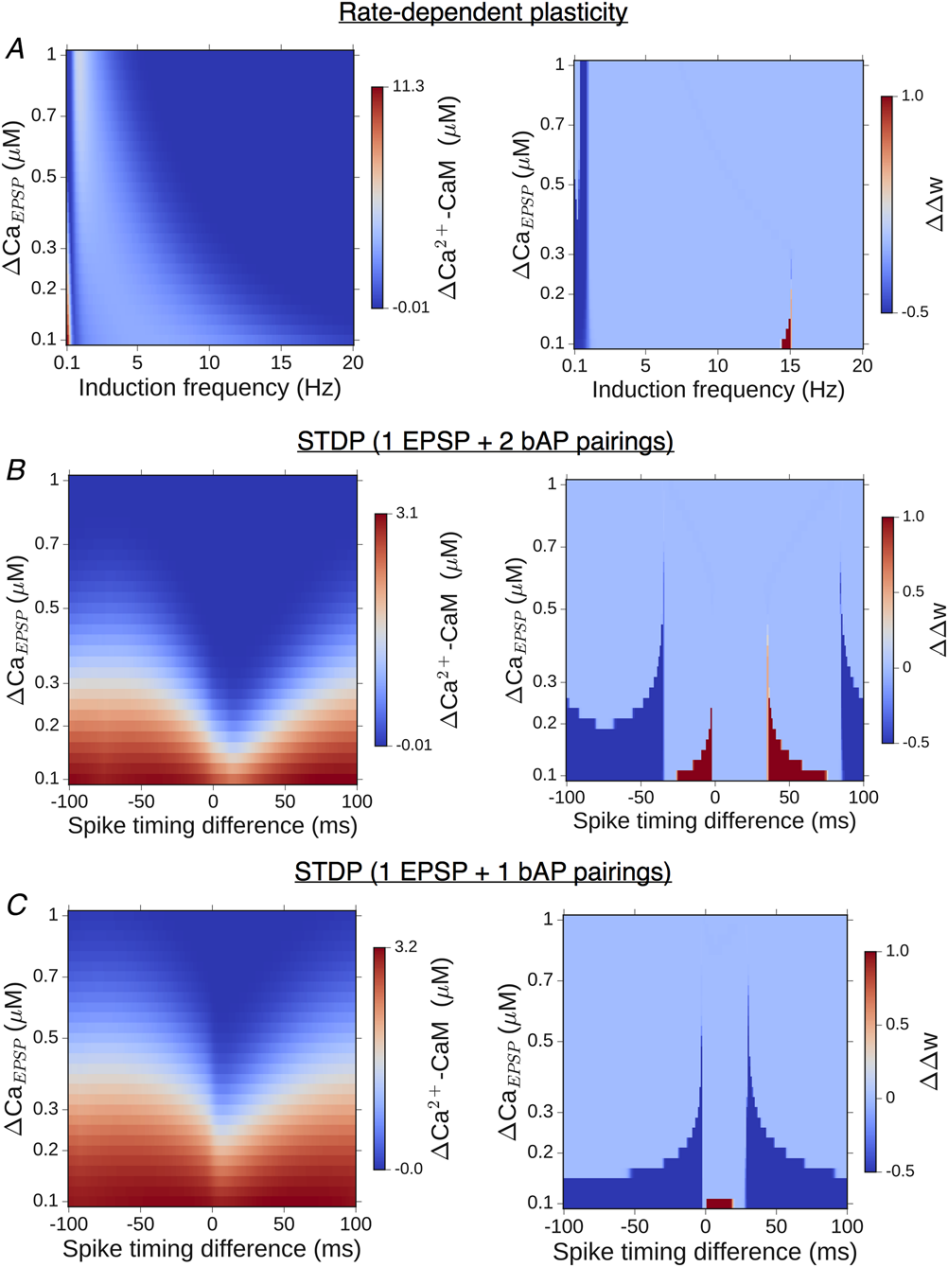
Differential enhancement of LTD and LTP by spine ER is a general consequence of ICCR kinetics *A*, left, change in maximum CaM activation due to the presence of ER as a function of the spine NMDAR conductance and induction frequency; right, dependence of the modified plasticity (change in Δ*w*) due to presence of ER on the NMDAR conductance and induction frequency. *B* and *C*, parameter sensitivity in the case of STDP inputs for the triplet (*B*) and doublet (*C*) spike pairing stimuli. (All results for comparison between ER^−^ control spine and an equivalent ER^+^ spine with 30 IP_3_Rs; plasticity thresholds appropriately adjusted to give (in the ER^−^ spine) *f*_D_ = 1 Hz and *f*_P_ = 15 Hz for rate‐dependent plasticity, and, separately, STDP for −35 ms < Δ*t* < 70 ms.) [Color figure can be viewed at wileyonlinelibrary.com]

Figure 9 *A* summarizes the differential responses in the ER^+^ spine (for *N*_R_ = 30 IP_3_Rs) relative to the ER^−^ (control) spine during trains of synaptic (SC) input at different rates. The heat map in Fig. 9 *A* (left) represents the excess Ca^2+^–CaM amplitude as a function of the input frequency (horizontal axis) and *g*_N_ (vertical axis). ER contributes robustly to spine Ca^2+^ elevation at low input frequencies (*f* ≲ 5 Hz). The contribution of ICCR steadily declines at higher input rates due to Ca^2+^‐dependent inhibition of IP_3_ receptors, and this frequency‐dependent suppression of the difference between the ER^−^ and ER^+^ spines is more pronounced at higher ΔCa_EPSP_. The corresponding results for the differential induction of plasticity in the ER^+^ spine are displayed as a heat map in Fig. 9 *A* (right). For each *g*_N_, the plasticity thresholds θ_D_ and θ_P_ have been adjusted to have *f*_D_ = 1 Hz and *f*_P_ = 15 Hz in the ER^−^ spine, and the excess plasticity (ΔΔ*w*) in the presence of ER has been estimated for every input frequency. Figure 9 *A* (right) indicates that ICCR robustly augments NMDAR‐mediated Ca^2+^ responses to facilitate the induction of synaptic depression at low input frequencies (*f* ≲ 1 Hz). Due to the inhibition of ICCR with increasing NMDAR activation (Fig. 9 *A*, left), there is little difference between the plasticity curves for the ER^−^ and ER^+^ spines at higher input frequencies. Except in a limited range of small *g*_N_ values (ΔCa_EPSP_ ≲ 0.15 μm), ICCR is strongly suppressed at stimulation rates above ∼10 Hz, and the LTP threshold (*f*_P_) in the ER^+^ spine remains nearly unchanged relative to the ER^−^ spine.

We similarly characterized the differential responses and plasticity outcomes in the ER^+^ spine during STDP input patterns (Fig. 9 *B* and *C*). For each *g*_N_, the plasticity thresholds θ_D_ and θ_P_ in our model were adjusted so as to yield (for spike triplets) an LTD window for −35 ms < Δ*t* < 0 ms and a potentiation window for 0 ms < Δ*t* < 35 ms in the ER^−^ spine. Figure 9 *B* (left) summarizes the dependence of the excess Ca^2+^–CaM response in the ER^+^ spine (with *N*_R_ = 30 IP_3_Rs) on *g*_N_ and the spike timing difference (Δ*t*) in the triplet case, showing a general reduction in the ICCR‐mediated enhancement of spine Ca^2+^ signals with increasing ΔCa_EPSP_. The corresponding differences in the plasticity outcome (ΔΔ*w*, Fig. 9 *B*, right) show significant broadening of the window of LTD induction over a fairly wide range of *g*_N_ values. The ER‐associated broadening of the LTP window is restricted to a comparatively narrow range of *g*_N_ values and spike timing intervals. The differential Ca^2+^–CaM response and plasticity outcome in the doublet case (Fig. 9 *C*) similarly highlight a general broadening of the window of spike timing differences eliciting synaptic depression, the extent of which scales inversely with ΔCa_EPSP_.

**Figure 10:**
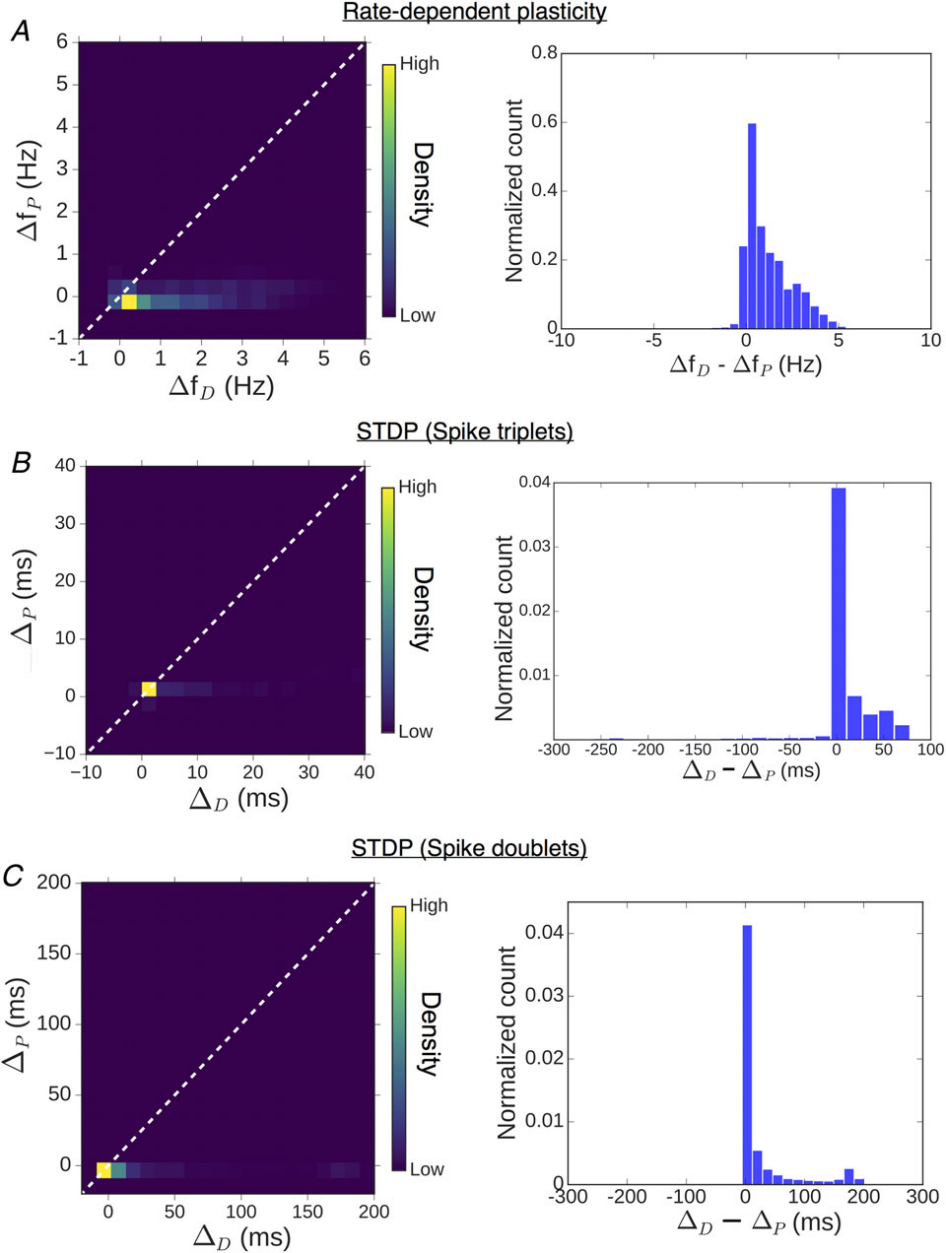
Differential enhancement of LTD and LTP by spine ER is broadly reproduced over a range of realistic parameter settings in our model Summary statistics of changes in plasticity windows introduced by ICCR in the case of frequency‐dependent plasticity (*A*), and for STDP with triplet (*B*) and doublet (*C*) spike pairing stimuli. In each case, the left figure is a heat map of the distribution obtained from random sampling of the thresholds at the ER^−^ control spine, over a range of IP_3_R cluster sizes (10–50) and NMDAR conductances (ΔCa_EPSP_ = 0.1–1 μM); the same information is represented as a 1D histogram of the relative differences (depression *vs*. potentiation) in the right figure. (For frequency‐dependent plasticity, *f*_D_ and *f*_P_ have been randomly sampled over 1–6 Hz and 10–20 Hz ranges, respectively. For STDP, Δ*t*_D_ and Δ*t*_P_ have been sampled from 20 ms windows centered on −35 ms and +35 ms, respectively.) [Color figure can be viewed at wileyonlinelibrary.com]

As the above results were obtained for a specific choice of the plasticity thresholds and IP_3_R cluster size, we repeated these comparisons with variable number of IP_3_ receptors (*N*_R_ = 10–50) and plasticity thresholds, for both frequency‐dependent plasticity and STDP (Methods). For each combination of parameter values (*g*_N_, *N*_R_, θ_D_, θ_P_), the overall modulation of the NMDAR‐only plasticity curve by ICCR was quantified in terms of the shifts in the LTD and LTP thresholds for rate‐dependent plasticity, and changes in the LTD/LTP window widths in the case of STDP. The summary statistics (Fig. 10 *A–C*) provide a sense of the variability in model outcomes, and taken together, suggest graded modulation of NMDAR‐based bidirectional plasticity by ICCR, with heightened LTD induction. In sum, our model simulations over realistic parameter ranges provide support for an ER‐linked form of synaptic metaplasticity on the level of individual spines, the nature of which is regulated by mGluR–IP_3_ signalling in concert with the temporal profile of NMDAR activation.

## Discussion

**Figure 11:**
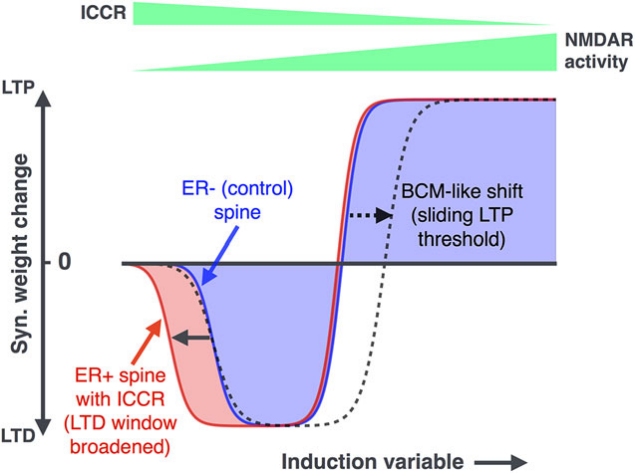
ER Ca^2+^ store introduces a novel form of synaptic metaplasticity at individual CA1 dendritic spines Results from our analysis of frequency‐ and spike timing‐dependent plasticity are summarized by a comparison of the plasticity profiles for the reference ER^−^ spine (blue) and a spine with ER (red). The induction variable (which can stand for the synaptic input frequency *f*, or spike timing difference Δ*t*) controls the activation of both NMDAR and ICCR, these dependencies being represented by tapering bars at the top. Also shown for comparison is the modified plasticity curve arising from a BCM‐like sliding LTP threshold (dashed curve). [Color figure can be viewed at wileyonlinelibrary.com]

Dendritic spines are specialized structures that facilitate spatially restricted biochemical signalling and enable input‐specific, Hebbian‐type synaptic changes mediated by NMDA receptors (Koch & Zador, 1993; Bourne & Harris, 2008). In the present study, we systematically investigated, with mathematical modelling, how the local reorganization of ER may modulate Ca^2+^‐driven plasticity on the scale of individual excitatory CA1 synapses. Results from our model simulations of rate‐based plasticity and spike timing‐dependent plasticity, taken together, suggest that the presence of ER selectively enhances the propensity for LTD induction with a relatively diminished effect on LTP induction. Targeting of ER to larger spine heads can thus mediate a ‘metaplastic switch’ at stronger synapses that specifically relies on ER Ca^2+^ handling and metabotropic glutamatergic signalling. The graded contribution of the IP_3_‐sensitive ER store to spine Ca^2+^ elevation as a function of NMDAR activation that we have characterized here offers a synapse‐specific mechanism to differentially modulate the windows for depression/depotentiation and potentiation, yielding a net enhancement of activity‐driven synaptic depression (Fig. 11). In light of the observed association of ER with more potent synapses (Cooney *et al*. 2002) and the dynamic acquisition of ER accompanying spine enlargement (Sala *et al*. 2005; Ng *et al*. 2014), we propose a regulatory role for ER as a compensatory ‘braking’ mechanism that may temper the propensity for further strengthening at the potentiated synapses. Our findings thus support a novel interpretation for spine ER in adjusting the plasticity profile of a synapse on an as‐needed basis, which may contribute to keeping saturation at bay and maintaining synaptic strengths within a useful dynamic range supporting memory storage and optimal neural network function (Abbott & Nelson, 2000; Abraham, 2008; Keck *et al*. 2017).

Synapse‐specific LTP in the hippocampal formation is essential for initiation of experience‐dependent learning (Kitamura *et al*. 2017). This locus is shifted subsequently and cortical neural circuits take over memory consolidation and retrieval after initial memory formation is completed (Kitamura *et al*. 2017; Eichenbaum, 2017). Spines having undergone experience‐dependent modifications can quickly saturate (runaway effect of LTP) and no longer participate in ongoing learning. Adaptability of the plasticity profile may promote re‐use of strong synapses, enabling saturated synapses to be brought back into the ‘game’. The form of metaplasticity elucidated here is specific to individual CA1 spines, and is likely triggered by the rapid local remodelling of ER that may be a consequence of strong prior synaptic activation (Ng *et al*. 2014). Our work is of particular relevance to an understanding of the mechanisms in place to overcome possible storage limitations in the CA1 field, given that ER is most likely to be found associated with stronger synapses at the larger CA1 spines. The selective enhancement of synaptic weakening in the presence of ER (Fig. 11) has a direct implication for long‐term stability of the stronger synapses: under ‘natural’ conditions (Klyachko & Stevens, 2006), background synaptic activation may promote a slow resetting of potentiated synapses at the ER‐bearing spines; viewed another way, acquisition of spine ER is anticipated to effectively ‘slow down’ cumulative synaptic strengthening over time. We note that these effects are distinct from a BCM‐like shift of the synaptic LTP threshold (Bienenstock *et al*. 1982), though at higher SERCA activity levels, ER may introduce such a shift in addition to promoting LTD induction (simulations not shown).

Computational models of Ca^2+^‐based plasticity normally attempt to link NMDAR‐mediated Ca^2+^ entry to downstream signalling events at the postsynaptic locus, and these have provided a meaningful account of the general phenomenology of long‐term plasticity in the hippocampus and neocortex (Zador *et al*. 1990; Shouval *et al*. 2002; Graupner & Brunel, 2007; Rackham *et al*. 2010; Kumar & Mehta, 2011; Graupner & Brunel, 2012). From a neurobiological point of view, though, it is important to go beyond averaged descriptions and consider whether local heterogeneities in microphysiology on the synapse level could introduce functional differences between individual synaptic connections in a population. The role of spine ER in introducing synapse‐specific functional differences in Ca^2+^ signalling may not be clearly discernible from macroscopic measurements of synaptic plasticity properties, or by lumping together and averaging over experimental data from many synaptic contacts only a few of which may be associated with ER‐bearing spines. This situation in hippocampal pyramidal neurons may be contrasted with the case of spines associated with cerebellar parallel fibre to Purkinje cell synapses, which are more homogeneous in terms of the presence of ER (Harris & Stevens, 1988) and where localized Ca^2+^ release from stores is known to be central to the induction of long‐lasting plasticity (Wang *et al*. 2000; Miyata *et al*. 2000; Antunes & De Schutter, 2012).

This last aspect, in particular, underlines the utility of a physiologically plausible *in silico* model which enables systematic characterization of the role of ER in Ca^2+^ signalling at individual spines. The framework presented here is one of the first to weave a quantitative description of metabotropic glutamate signalling into a detailed model of spine Ca^2+^ dynamics driven by NMDAR activation. Delineation of the role of ER Ca^2+^ handling in a spine requires considering mGluR–IP_3_ signalling in the backdrop of synaptic NMDAR activation, as NMDAR‐gated Ca^2+^ entry can regulate several aspects of this signalling, including PLCβ activity, IP_3_ turnover and IP_3_R gating, with dynamic Ca^2+^ feedback from ICCR further adding to the overall system complexity (Fig. 1 *C*). We calibrated the parameters in our model of ICCR to be consistent with salient features of experimentally reported Ca^2+^ responses in ER‐containing CA1 spines to unitary synaptic events (Holbro *et al*. 2009), particularly the lag (few hundred milliseconds) in Ca^2+^ release from ER following release of glutamate into the synaptic cleft. Our analysis builds on these observations as we examine the contribution of ER to the spine Ca^2+^ response, and its subsequent shaping of plasticity, in the context of neural activity patterns that mimic the experimental induction of early LTP/LTD.

The response of an ER^+^ spine in our model to low‐frequency glutamate pulses, in particular, highlights the interaction of multiple time scales associated with IP_3_ degradation, recovery of IP_3_Rs from Ca^2+^‐dependent inactivation, and the input frequency in shaping the profile of ICCR. Our model yields a non‐monotonic rate dependence of the ER Ca^2+^ contribution at low input frequencies (*f* ≲ 5 Hz) (Fig. 4 *D*). As noted before, this arises from a balance between the contrasting effects of the input rate on (1) recovery of IP_3_Rs from inactivation between inputs (which is lower, the more closely spaced the inputs are), and (2) the availability of IP_3_, which increases with input rate and promotes the opening of IP_3_R channels. Analogous to the profile of ER Ca^2+^ contribution, our model also predicts a non‐monotonic rate dependence for the time delay in ICCR following glutamate release, which initially increases before decreasing with the input frequency as ICCR gradually synchronizes with the NMDAR‐mediated Ca^2+^ transient. The foregoing properties of spine Ca^2+^ response as functions of the synaptic input frequency, inferred from our numerical simulations, are potentially informative readouts of the model which could be compared with similar readouts from Ca^2+^ imaging experiments at individual CA1 spines. Such a comparison would help validate the interplay of different kinetic time scales in the ER^+^ spine model used in this study and constrain key model parameters.

The contribution of ICCR to spine Ca^2+^ signalling can be broadly understood in terms of NMDAR‐mediated spine Ca^2+^ regulation and the kinetics of IP_3_R with its characteristic bell‐shaped dependence on the cytosolic Ca^2+^ level. With repeated low‐frequency stimulation, there is no sustained build‐up of Ca^2+^ evoked by the sequence of glutamate pulses. Every synaptic input triggers transient mGluR activation, evoking a short burst of Ca^2+^ release from ER which is initiated by fast activation (*m*_1_ and *m*_2_) of IP_3_R influx, followed by the slower Ca^2+^‐dependent inactivation (*h*) of the IP_3_R which brings the Ca^2+^ level down. This allows the IP_3_R to recover from inactivation between successive inputs when Ca^2+^ has decayed back to near‐resting levels, and explains the robust augmentation of the NMDAR‐mediated Ca^2+^ signal by ICCR at low stimulation frequencies (Fig. 4). At higher input frequencies, NMDAR‐gated Ca^2+^ transients triggered by successive inputs add up, and there is sustained elevation of Ca^2+^ in the spine, the magnitude of which is set by the input frequency *f*. As Ca^2+^ is continually maintained at a high level, the Ca^2+^‐dependent inactivation variable *h* stays small, and keeps the IP_3_R persistently inhibited as long as the stimulation is present, despite the concurrent activation of mGluR and availability of IP_3_. This underlies the progressive frequency‐dependent suppression of Ca^2+^ flux through IP_3_ receptors at higher input frequencies (Fig. 5), and implies a steadily diminishing role for ICCR as LTD switches to LTP according to the Ca^2+^‐based plasticity model governing bidirectional synaptic strength changes (Fig. 6 *D* and *E*).

An analogous situation arises during trains of pre‐ and postsynaptic spikes at a constant rate (in the theta band), mimicking the experimental induction of spike timing‐dependent plasticity. The activation of NMDARs in this case is controlled by the relative timing of pre‐ and postsynaptic firing (Δ*t*) on the millisecond scale. The amplitude of the sustained Ca^2+^ elevation driven by NMDA receptor activity is thus a function of the spike timing difference, and it directly controls the extent of inhibition (*h*) of the IP_3_R (Eq. 7). This accounts for the spike timing dependence of the relative contribution of ICCR to the total spine Ca^2+^ signal in Fig. 7, and the overall enhancement of synaptic depression with the addition of ER (Fig. 8 *C* and *D*).

The key findings following from our simulations are fairly general, and hold for variation in important model parameters such as NMDAR conductance and number of IP_3_Rs within realistic ranges. Other parameters, such as those controlling the spine–dendrite coupling (electrical and diffusional), were kept fixed based on literature estimates; however, our results are not sensitive to these specific values and higher values yield similar plasticity profiles. Further, our main conclusions regarding the contribution of ER to spine Ca^2+^ dynamics are also valid when IP_3_Rs are modelled stochastically to account for fluctuations arising from small numbers of receptors.

Regulation of synapse strength is acknowledged to be a complex process, likely involving the coordinated action of several mechanisms that may act over a wide range of time and spatial scales to modulate the rate and direction of ongoing activity‐dependent plasticity. Previously proposed mechanisms include intrinsic plasticity of membrane excitability (Narayanan & Johnston, 2010; Lee & Chung, 2014), compensatory scaling of spine volume to balance synaptic potentiation (Kalantzis & Shouval, 2009; O'Donnell *et al*. 2011), alterations in the number and/or subunit composition of postsynaptic NMDARs (Xu *et al*. 2009; Lee *et al*. 2010), and global synaptic scaling mediated by glial signals (Stellwagen & Malenka, 2006). The present study linking postsynaptic ER to local modulation of Ca^2+^‐based plasticity adds to the repertoire of dynamically regulated biophysical mechanisms that may be in place to control plasticity and function at excitatory hippocampal synapses (Zenke & Gerstner, 2017; Keck *et al*. 2017).

Although several lines of evidence link group I mGluR activation and ER Ca^2+^ stores to long‐term depression at hippocampal synapses, the involvement of ER, or more generally of mGluR signalling, in synaptic potentiation is less clear. Results from several pharmacological and knockout studies collectively do not implicate an essential role for group I mGluR in LTP or AMPAR‐mediated synaptic transmission in the CA1 region (Bortolotto *et al*. 1999). Mutant mice lacking a function G protein associated with group I mGluRs were found to be deficient in hippocampal LTD induced by low‐frequency stimulation, but exhibited intact LTP in response to tetanic inputs (Kleppisch *et al*. 2001). Some studies on mGluR5 knockout mice reported reduced LTP in CA1 neurons (Lu *et al*. 1997; Jia *et al*. 1998), but this was shown to involve selective reduction of NMDAR function on both the induction and expression levels, with no effect on the AMPAR component of LTP. Further, there is no direct evidence that the above interaction relies on Ca^2+^ release from stores, or that it is synapse‐specific and restricted to spines containing ER. As noted previously, LTP studies usually examine plasticity on the coarse‐grained level and not at individual synapses; thus, they are of limited utility in addressing the differences in local Ca^2+^ signalling that might arise between ER^−^ and ER^+^ spines during LTP induction. Moreover, the concurrent stimulation of multiple synaptic contacts (as in experimental induction of plasticity) may evoke Ca^2+^ release from ER in the dendritic body as well (Nakamura *et al*. 1999); this could activate signalling pathways distinct from those associated with early LTP in spines, and trigger plasticity at a large number of synapses (spread out over a wider dendritic region), independent of their association with ER‐bearing spines. The present study is restricted to quantifying the contribution of ER (IP_3_‐sensitive stores) to spatially confined Ca^2+^ dynamics in spines, and it is in this specific context that our analysis predicts decreasing ER contribution with stronger synaptic (NMDAR) activation due to the Ca^2+^‐dependent suppression of IP_3_R activity. We note that an early experimental study of STDP at CA3–CA1 synapses (Nishiyama *et al*. 2000) makes a similar suggestion about the suppression of IP_3_R activity during LTP (but not LTD) induction, as a possible explanation for the absence of associated heterosynaptic plasticity mediated by regenerative Ca^2+^ release (Ca^2+^ waves) from IP_3_‐sensitive stores. The predicted graded effect of spine ER on plasticity curves could be potentially tested by imaging spine Ca^2+^–CaM activity during different forms of persistent stimulation at individual CA3–CA1 synapses contacting ER^+^ spines, and assessing the effect of pharmacological blockade of either stores (SERCA inhibitors such as thapsigargin) or specific inhibition of the IP_3_ pathway (e.g. with heparin) on their response profiles. Such a comparison should ideally control for the possible confounding effects of changes in spine volume/synapse strength that may concurrently occur due to activity‐induced plasticity, through targeted inhibition of key effectors downstream of CaM, e.g., CaMKII and calcineurin.

To conclude, we have presented a detailed modelling study, leveraging previous experimental observations, to characterize the local modulation of Ca^2+^ signals and plasticity at hippocampal dendritic spines by ER. Our analysis quantifies a unique role for ER in dynamically altering the long‐term plasticity profile at individual CA1 spines and is supported by experimental evidence (Sala *et al*. 2005; Holbro *et al*. 2009; Ng *et al*. 2014). Our study makes general predictions about a differential effect of ER on LTD and LTP under physiological conditions that is qualitatively distinct from BCM‐like mechanisms, besides providing detailed quantitative characterization of ER Ca^2+^ regulation via metabotropic signalling as a function of the temporal profile of inputs and synaptic NMDAR activation patterns. The synapse‐specific role of ER suggested here adds a new dimension to earlier work on metaplasticity mediated by metabotropic receptors (Cohen & Abraham, 1996; Ireland & Abraham, 2002; Bortolotto *et al*. 2005; Abraham, 2008). Incidentally, a role for store Ca^2+^ release was also recently implicated in synaptic homeostasis mediated by spontaneous miniature EPSPs (Reese & Kavalali, 2015). The involvement of ER (as a Ca^2+^ store) in biochemical signalling likely extends to other aspects of dendrite function, such as in mediating some forms of heterosynaptic plasticity (Nishiyama *et al*. 2000), long range (synapse‐to‐nucleus) signalling involved in activity‐dependent transcriptional regulation (Bading, 2013), and spatiotemporal integration of synaptic inputs (Lee *et al*. 2016); recent studies also link perturbations of the pathways considered here to early pathological events reported in some forms of Alzheimer's disease and fragile X syndrome (Bear *et al*. 2004; Nakamoto *et al*. 2007; Cheung *et al*. 2008; Pchitskaya *et al*. 2018). The approach presented here provides a framework for future modelling studies aimed at investigating these diverse questions. On a more general note, the potential contribution of ER to microscale signalling elucidated here highlights the need to move beyond ‘average’ accounts of synaptic signalling, and consider the implications of local physiological differences between synapses for their plasticity and function.

## Additional information

### Competing interests

The authors declare that no competing interests exist.

### Author contributions

G.M. and S.N. conceived the study and designed the experiments. G.M. performed the experiments and analysed the data; G.M. and S.N. wrote the paper. All authors have read and approved the final version of this manuscript and agree to be accountable for all aspects of the work in ensuring that questions related to the accuracy or integrity of any part of the work are appropriately investigated and resolved. All persons designated as authors qualify for authorship, and all those who qualify for authorship are listed.

### Funding

S.N.: Wellcome Trust/DBT India Alliance (grant number IA/I/12/1/500529). G.M.: Wellcome Trust/DBT India Alliance (grant number IA/I/12/1/500529) and Science & Engineering Research Board, India (grant number PDF/2017/001803)
